# Assessing graphite schist as a supplementary cementitious material in concrete for antifungal activity, strength, hydration, microstructure, and radiation shielding

**DOI:** 10.1038/s41598-026-40900-0

**Published:** 2026-04-09

**Authors:** Mostafa Serry, A. M. Zayed, Aya I. Tagyan, Wageeh Ramadan, Bahaa S. Metwally, Alaa M. Rashad, Hussain Shendy, Ahmed M. A. Abdelgwad, Mohamed H. Abdelaziz, A. M. El-Khayatt, W. A. Kansouh, M. G. Shahien, M. A. Masoud

**Affiliations:** 1 Applied Mineralogy and Water Research Lab (AMWRL), Geology Department, Faculty of Science, Beni-Suef University, Beni Suef, 62521 Egypt; 2 Department of Botany and Microbiology, Faculty of Science, Beni-Suef University, Beni-Suef, 62521 Egypt; 3 Radiation Protection and Safety Department, Hot Laboratories and Waste Management Center, Egyptian Atomic Energy Authority (EAEA), Cairo, 13759 Egypt; 4 Textile Technology Department, Faculty of Technology and Education, Beni-Suef University, Beni-Suef, 62521 Egypt; 5 Civil Engineering Department, College of Engineering, Shaqra University, Al-Dawadmi, Riyadh Saudi Arabia; 6 Building Materials Research and Quality Control Institute, Housing and Building National Research Center (HBRC), Cairo, Egypt; 7 Department of Physics, College of Science, Imam Mohammad Ibn Saud Islamic University (IMSIU), Riyadh, Saudi Arabia; 8 Nuclear Research Center, Egyptian Atomic Energy Authority (EAEA), Cairo, 13759 Egypt

**Keywords:** Concrete, Cement, Isothermal calorimetry, Metamorphic rock, SCM, Engineering, Materials science

## Abstract

For the first time, this study explores the valorization of graphite schist (GS), a metamorphic rock, as a novel supplementary cementitious material (SCM) in concrete. Portland cement (PC) was partially replaced with GS at 0, 10, and 15% by mass to prepare CC (control), GC10, and GC15 mixes, respectively. Physical, mechanical, hydration, microstructural, and radiation shielding properties were investigated. GS showed superior particle fineness, higher surface area, notable antifungal activity, and remarkable thermal stability up to 800 °C, outperforming PC in several aspects. Unlike GC15, GC10 achieved sustained compressive strength, closely related to that of CC. Microstructural analysis revealed that GS functioned mainly as a filler with limited pozzolanic reactivity, contributing moderately to particle packing. However, unhydrated cement particles and weakened interfacial transition zones were more prevalent at 15% GS. The improved performance of GC10 over curing periods reflected the more favorable influence of 10% GS on hydration and microstructure. Moreover, GS incorporation slightly improved fast neutron attenuation but reduced gamma-ray shielding due to increased porosity and reduced density. Overall, GS can be a promising multifunctional SCM, offering antifungal potential, acceptable neutron shielding, balanced strength, and reduced PC usage at 10% replacement.

## Introduction

Developed countries are actively working to alleviate the influences of climate change by lowering CO_2_ emissions as a major reason for this global issue. CO_2_ emissions significantly contribute to the greenhouse effect by trapping the thermal radiation in the atmosphere, thereby accelerating global warming. Over the past two centuries, CO_2_ levels have risen sharply because of the industrial development. The cement industry is among the greatest suppliers of CO_2_ emissions, primarily due to its intensive energy consumption and high production costs^1^. Among all construction materials, concrete consumes the highest volume of cement. Cement production alone is a substantial source of greenhouse gases, contributing to nearly 7–8% of global yearly anthropogenic CO_2_ emissions^2^. Concrete is not only applied in construction works but also in radiation shielding^3–5^, suppressing other materials such as glass and polymers^6–8^. Recently, radiation shielding has become a major protective factor in the development of nuclear reactors for electrical power generation and radiotherapy centers. Therefore, the effective shielding materials are essential for reducing harmful radiation exposure to acceptable limits to protect the living organisms.

As a partial substitute for clinker, supplementary cementitious materials (SCMs) offer a promising strategy to alleviate CO_2_ emissions associated with cement production. More specifically, substitution of 1000 g of clinker with an equivalent amount of SCMs can put off emission of 1000 g of CO_2_^9^. Moreover, SCMs contribute to diminishing industrial waste and upgrading the different properties of concrete, like durability and microstructure^10–12^. From a microstructure, SCMs can play a key role in refining pore structure, improving durability, and strengthening interfacial transition zone (ITZ) between binder and aggregates^13–15^. Proficiency of SCMs largely depends on their content of amorphous (reactive) silica, which is principally responsible for driving the pozzolanic reaction with Ca(OH)_2_ (portlandite). This reaction results in generating calcium silicate hydrate (CSH), the principal hydration product that enhances concrete strength. Moreover, SCMs upgrade the microstructure, reduce permeability, and enhance chemical resistance to acids and sulfates in cementitious materials^16^. Based on their origin, SCMs can be broadly categorized into two categories: those derived from natural sources and those obtained as industrial by-products. Natural SCMs include volcanic ash^17^, limestone^18^, mica, feldspars^19^, and diatomite^20^. In fact, the industrial waste can be malignant solid by-products, increasing the environmental burden. Alternatively, such waste includes fly ash^21^, slag^22^, silica fume^23^, brick waste^24^, and glass waste^25,26^. Moreover, other effective SCMs were developed by the semi-wet carbonation of minerals such as wollastonite^27,28^. Incorporating such materials into concrete or mortar not only lowers the environmental footprint of construction but also boosts performance characteristics. Furthermore, recent studies explored the influence of some SCMs on radiation shielding of concrete^29–31^.

Despite the growing body of literature on SCMs, numerous minerals and rocks remain underexplored and warrant further investigation. Schists are one of these rocks that have a wide variation of mineral composition, but there is a deficiency in the studies of their engineering, antimicrobial, and radiation attenuation properties. Schists are medium- to coarse-grained metamorphic rocks that originated from metamorphism of sedimentary rocks (e.g., shale, sandstone, limestone), but with preservation of some relics such as layering, so they are characterized by foliation^32,33^. Schists vary in mineral composition, typically including mica and chlorite, with occasional presence of garnet, quartz, graphite, talc, or amphiboles (e.g., hornblende, actinolite, or tremolite)^33^.

Like geology, schists can be imperative in engineering applications due to their miscellaneous mineral content, chemical composition, and mechanical properties^34,35^. Schists have been investigated for various applications, including ornamental stones^36^, aggregates in concrete^37^, and road pavement materials^38^. More specifically, their rich mineral content, as previously mentioned, contributes to a chemical composition that includes silica, alumina, carbon, iron, and crystalline water. These components are critically necessary for both the cement industry and radiation shielding applications^39–41^. Therefore, some studies have explored the incorporation of schists in radiation shielding Portland cement clinker by sintering at 1450 °C^42^. Moreover, schists have been previously evaluated as SCMs^43,44^. Although schist rocks are enriched in SCM-related oxides such as SiO_2_, Al_2_O_3_, CaO, and Fe_2_O_3_, similar to those found in clay minerals, their potential as radiation shielding SCMs has not been extensively investigated. Based on the available literature of SCMs, their wide-ranging mineralogical and chemical composition, antimicrobial activity, thermal stability, and hydration evolution remain largely unexplored. Additionally, their influence on the microstructural properties of cementitious systems has not been deeply discussed.

More specifically, the Abu Fannani schist (Abu Fannani thrust sheet) basement unit is prominently exposed in the Meatiq dome, Eastern Desert of Egypt^38^. This rock unit is part of a sequence of highly deformed and metamorphosed rocks ranging from greenschist to amphibolite facies. Abu Fannani schist has variance in composition, including biotite-garnet schist, hornblende biotite schist, and graphite schist with subordinate amphibolite^45^. Particularly, the graphite schist (GS), compared to other schist types, is primarily composed of actinolite-tremolite, hornblende, graphite, quartz, plagioclase, and biotite with a reasonable presence of iron oxides. Due to the variation in mineral and chemical composition of GS, remarkably the content of iron oxides, graphite, and crystalline water, GS can be potentially qualified for radiation shielding compared to other schist types like quartz, garnet, or mica schists. Accordingly, our study aims to evaluate the potential use of GS from the Abu Fannani unit as SCM in concrete, with particular emphasis on its impact on radiation shielding performance. Therefore, the study focuses on assessing the effects of incorporating GS at varying replacement ratios (10% and 15% by mass of PC) on the physical, mechanical, hydration, microstructural, and radiation shielding characteristics of the resulting concrete. Additionally, the antifungal properties of GS were considered as part of the evaluation. This was implemented through partially substituting PC with 10 and 15% of GS by weight and then studying their impact on the previously mentioned characteristics of concrete and PC.

## Material and methods

### Material collection and preparation

**Fig. 1:**
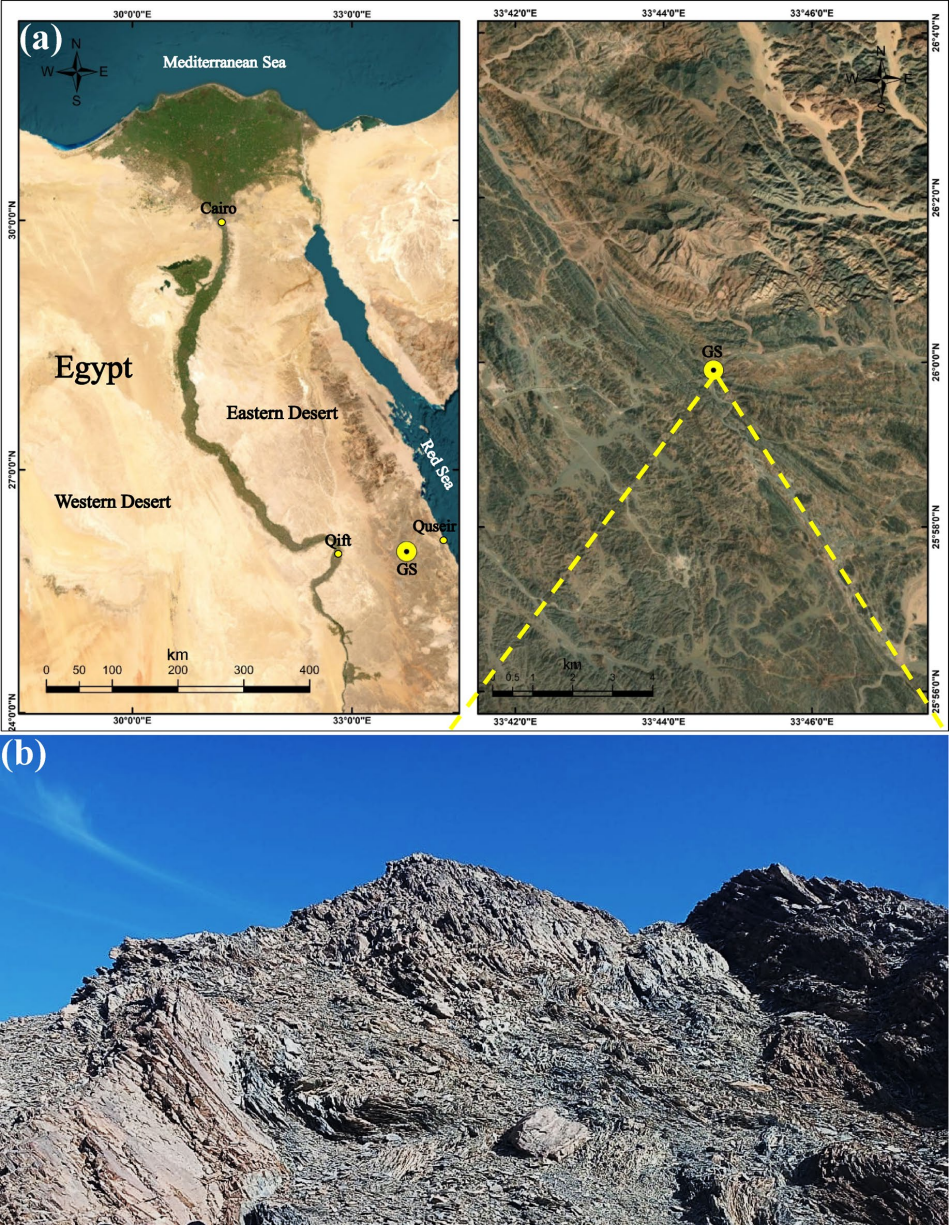
(**a**) Location map generated using ArcGIS Desktop version 10.8 (ArcMap, Esri, Redlands, CA, USA; https://www.esri.com), showing the sampling location of GS along the Qift-Quseir Road, Eastern Desert, Egypt; (**b**) Field photograph showing folded and foliated outcrops of Abu Fannani schist, from which the GS sample was collected.

**Fig. 2:**
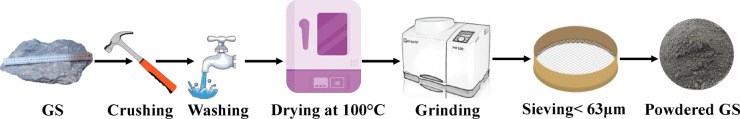
Preparation procedure of GS before blending with PC.

**Fig. 3:**
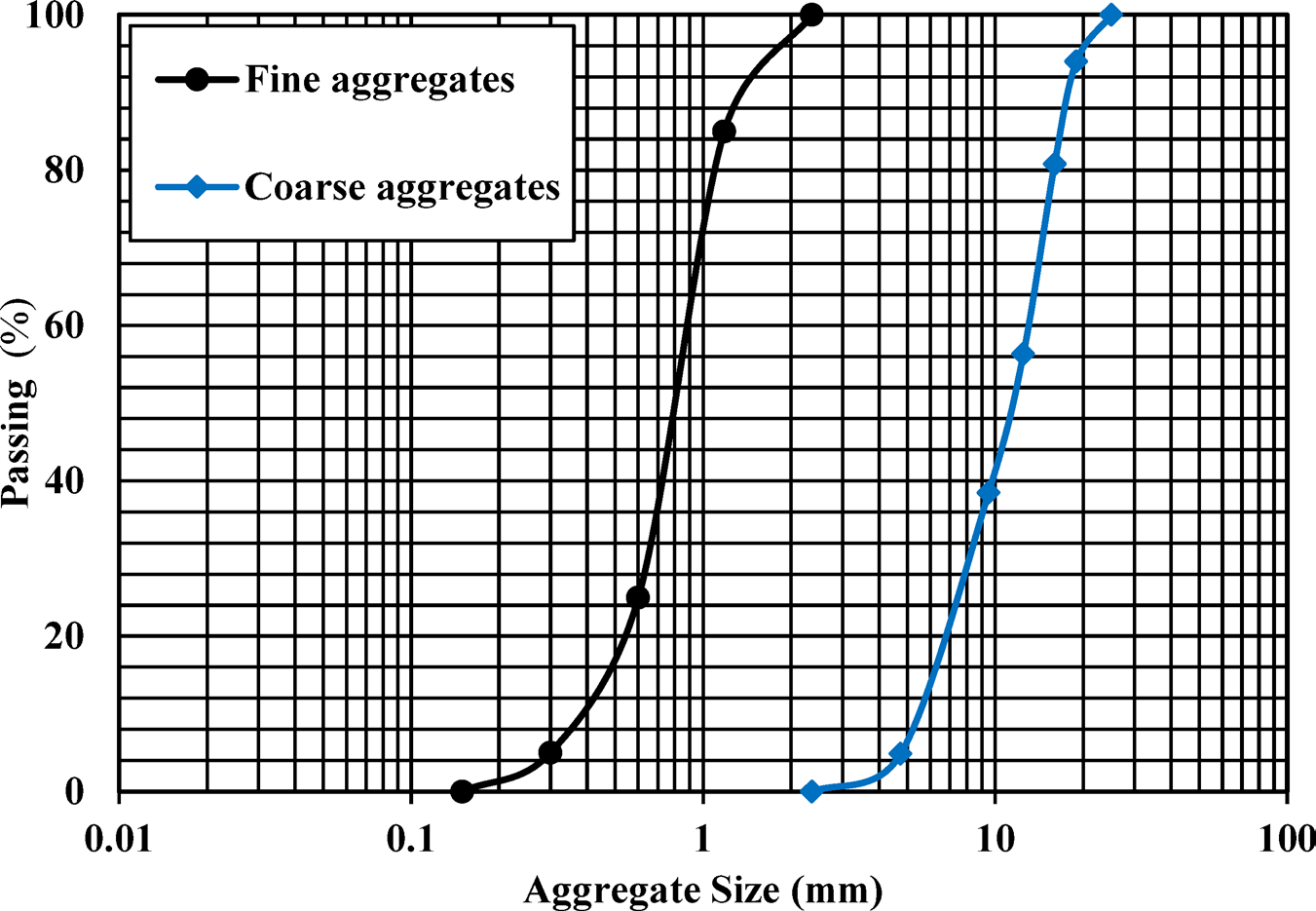
Particle size distribution profiles of aggregates used in concrete mixes.

The utilized sample of graphite schist (GS) used in this study was obtained from the Abu Fannani schist unit, located in the Meatiq dome area along the Qift-Quseir Road, Eastern Desert, Egypt (Fig. 1a). As shown in Fig. 1b, GS sampling was deliberately collected from structurally deformed zones exhibiting prominent foliation and folding to induce an effective crushing process. As illustrated in Fig. 2, the GS sample was initially crushed using a geological hammer to obtain fragment fractions within 5–10 mm. Then, this crushed GS was cleaned using tap water to eliminate any detrimental materials, such as clay or gypsum. After that, the sample was dried in an oven at 100 °C for 24 h. Final grinding was performed using a planetary ball mill (RETSCH, PM100, UK) for 20 min, and the resulting material was sieved to a particle size of < 63 μm (Fig. 2). Concrete ingredients used in this study included dolomitic limestone (coarse aggregate), sand (fine aggregate), PC (CEM I, 42.5 N/mm^2^), and potable water. According to ASTM C33^46^, the coarse and fine aggregates have particle size distributions shown in Fig. 3.

### Material characterization

#### Physical characterization

Employing the volumetric cylinder of 1 L (1000 cm^3^), the bulk density of PC and powdered GS samples (< 63 μm) was characterized, following the equation:

$$\rho = w/v$$

where *ρ* represents density (g/cm^3^), *w* denotes material mass (g), and *v* signifies the cylinder volume (1000 cm^3^).

The measurement of PC and GS bulk density largely depends on powder handling, whether it is poured freely, tamped, tapped, or compacted. Following the method described by Ogbodo and Akpabot^47^, each of PC and GS was separately poured into the volumetric cylinder in three layers, followed by tapping for each layer. Excess powder at the top of the cylinder was then levelled using a straightedge. All measurements were performed in triplicate for each powder type.

#### Mineral and chemical composition

The previously prepared powdered sample of GS (< 63 μm) and PC were subjected to X-ray diffraction (XRD) analysis, using an Empyrean diffractometer (Malvern Panalytical BV, Almelo, The Netherlands). Scans were performed between 5 and 80° (2θ), applying a step size of 0.04° and a counting time of 0.5 s/step in continuous mode.

As outlined in ASTM E1621 and D7348^48,49^, the powdered GS and PC were analysed by X-ray fluorescence (XRF) to detect its elemental composition.

#### Particle size distribution (PSD)

According to ISO 13320^50^, the particle size distribution (PSD) of the PC and GS samples was determined over a measurement range of 0.01–2100 µm, using laser diffraction analysis (LDA). Measurements were performed using the ANALYSETTE 22 MicroTec Plus instrument (Fritsch GmbH, Germany).

#### BET surface area

To detect the specific surface area of PC and GS, the Brunauer–Emmett–Teller (BET) technique was conducted, employing the N_2_-adsorption–desorption isotherm at − 196 °C. This was performed using the Quantachrome Autosorb iQ automated gas adsorption system (Quantachrome Instruments, USA)^51^.

#### Morphological features

For distinguishing the morphological nature of PC and GS, their powdered samples were analyzed by a field emission scanning electron microscope (FE-SEM, Quanta FEG 250, FEI Company, USA). Before imaging, the samples were layered with an Au nanofilm (~ 8 nm) to ensure sufficient surface conductivity.

#### Thermal stability

Thermogravimetric analysis (TGA) and derivative thermogravimetry (DTG) were performed for GS material to evaluate the thermal stability and decomposition performance. They were conducted by an SDT Q600 V20.9 Build 20 thermal analyzer (TA Instruments) under a N_2_ atmosphere with a temperature range from ambient to 800 °C at a heating rate of 10 °C/min and N_2_ flow rate of 50 ml/min.

#### Hydration evolution (isothermal calorimetry)

Employing isothermal calorimetry, the hydration mechanism of GS in the cementitious pastes was studied by attaining their heat release for 48 h. During the analysis process, a water-to-cement ratio (w/c) of 0.50 was maintained, with PC partially replaced by 10% and 15% GS, by mass. For 4 min, approximately 10 g of each sample was hand mixed. Subsequently, 7 g of paste was transferred into a sealed ampoule and put into the calorimeter, maintained at the room temperature (22.50 °C). The first 60 min of calorimetry data were disregarded from the output data owing to initial temperature fluctuations^52,53^.

#### Antifungal activity

The antifungal susceptibility of PC and GS was evaluated for six fungal strains of filamentous and yeast types: *Aspergillus flavus (A. flavus*), *Aspergillus niger (A. niger)*, *Aspergillus lentulus (A. lentulus)*, *Candida albicans* (*C. albicans*), *Curvularia lunata (C. lunata)*, and *Fusarium oxysporum (F. oxysporum).* For performing this test, the agar well diffusion method was utilized. This method is a common technique for detection of antifungal screening^54^. The samples of PC and GS were dissolved in a solvent composed of equal parts ethanol and distilled water to facilitate solubility, yielding a stock solution of 100 mg/mL. To ensure the purity of spore suspensions, fungal spores (10⁶–10⁷ spores/mL) were suspended in sterile 0.1% Tween-80 and filtered to dispose of mycelial remnants. Plates of potato dextrose agar were regularly inoculated with the fungal suspension by sterilized swabs. Holes with 6 mm diameter were then punched aseptically, using a sterilized cork borer^55^. After that, 100 µL of the sample solution (GS or PC) was introduced into each hole, while the control hole received only the solvent mixture (negative control). These plates were then subjected to incubation at 25–30 °C for a period of 24–48 h. Following incubation, inhibition zones (mm) were valued in three perpendicular directions and averaged to measure antifungicidal efficacy quantitatively. All experiments were independently replicated three times to support reproducible and accurate outcomes.

### Concrete mix proportions

As specified by ACI 211^56^, the concrete mix designs were developed per 1 m^3^ of normal concrete, as detailed in Table 1. The w/c, along with the fine and coarse aggregates, was kept unchanged across all mixtures. The only variable parameter was the replacement ratio of PC by GS. Namely, the control concrete mix (CC) consisted entirely of 100% PC, while the modified mixes, GC10 and GC15, included 10% and 15%, respectively, representing increasing levels of PC replacement by GS.

**Table 1:** Concrete mix design (ingredients in kg/m^3^).

Concrete	Coarse agg	Fine agg	PC	GS	w/c
CC	1155	770	350	–	0.50
GC10	1155	770	315	35	0.50
GC15	1155	770	297.50	52.50	0.50

### Concrete preparation

Initially, limestone and sand aggregates were submerged in water for about 2.5 h to diminish the absorption of mixing water^57^. Afterwards, they were dispersed on the ground to reach a saturated surface dry (SSD) state. For CC, aggregates and PC were firstly dry mixed for 30 s by a rotating drum mixer. Water was then gradually added over another 3 min, followed by an additional 30 s of mixing to ensure full homogeneity, yielding a total mixing time of 4 min to guarantee the mix homogeneity. Prolonged mixing time was deliberately prevented to avoid any aggregate segregation. Prior to preparing the GC10 and GC15 concrete mixes, PC was thoroughly blended with GS at replacement levels of 10% and 15% of weight, respectively, as presented in Table 1. These mixes were fabricated following the same mixing procedure as CC mix. Fresh concrete was cast in oiled iron molds of cubic (10 cm) dimensions, as stipulated in ASTM C192^58^. Proper compaction was conducted using a vibrating table to eliminate entrapped air and minimize voids. Following 24 h of casting, the specimens were demolded at room temperature and cured in water. The curing regime involved intervals of 3, 7, 14, 28, 56, 90, and 180 days to monitor the strength development over time.

### Concrete characterization

#### Workability and physico-mechanical characterization

As specified in ASTM C143^59^, workability of all fresh concrete was evaluated by slump test. After 28 days of curing, the physical properties of concrete specimens, including density and water absorption, were evaluated as described in ASTM C642^60^. Additionally, the porosity was measured according to the calculation as described in the preceding literature^61,62^. Triplicate measurements were conducted, and the corresponding average values were recorded.

Concerning the strength tests, the compressive strength (*f*_*c*_) was measured in compliance with BS EN12390-3^63^. *f*_*c*_ was measured under a loading rate of 0.60 MPa/s, utilizing a compression testing machine with 2000 kN as a maximum capacity. *f*_*c*_ was assessed at 3, 7, 14, 28, 56, 90, and 180 days for both control concrete (CC) and the optimum GS-blended mix (either GC10 or GC15), which exhibited comparable *f*_*c*_ values to the CC at 28 days. For accuracy and reliability, each test was executed in triplicate at every curing period, and the average of the three measurements was documented.

#### Hydration and microstructure characterization

Understanding the hydration process and microstructural development of concrete is essential for evaluating the effectiveness of SCMs and their influence on both mechanical strength and durability^13,64,65^. Accordingly, three concrete samples were selected for further microstructural analyses: the control sample (CC), the most effective replacement level, and a higher GS content sample. Following 28 days of curing, as outlined earlier, XRD analysis was implemented for the powdered samples (< 63 µm) to characterize the hydration products and residual unreacted cement phases. For further corroboration, these powdered samples were also inspected by Fourier transform infrared spectroscopy (FTIR) to underline the existence of both hydrated and unhydrated phases. FTIR was performed over 400–4000 cm^−1^ at a resolution of 4 cm^−1^ to reveal the functional groups, characterizing these hydrated and unhydrated phases.

For clear visualization of the microstructure of concrete samples evidently, they were prepared by a detailed and controlled polishing process. After 28 days of curing, thin sections of each specimen (25 mm × 25 mm × 5 mm) were cut perpendicular to the casting direction using a precision saw. Afterwards, these section surfaces were leveled to certify uniform electron interaction during SEM inspection. Subsequently, they were subjected to a multi-step polishing procedure to enable high-resolution imaging of the microstructural features, with particular emphasis on the ITZ^66,67^. The first polishing stage involved sequential polishing by 11 waterproof silicon carbide (SiC) abrasive papers of grit numbers of 60, 120, 320, 600, 800, 1200, 1500, 2000, 2500, 3000, and 5000. Through this stage, anhydrous ethanol was used as a lubricant and coolant, as water could interfere with secondary hydration or ongoing curing. This was followed by the second stage, which involved four oil-based diamond polishing pastes with particle sizes of 2.50, 1.50, 1.00, and 0.50 µm. Following this, the specimens were oven-dried for 24 h at 65 °C. As outlined earlier, imaging was performed on the dried polished samples using a FE-SEM (Quanta FEG 250, FEI Company, USA), after gold coating. SEM imaging was executed in backscattered electron (BSE) mode at a constant magnification to allow reliable comparisons of morphological features and pore structure. Moreover, an energy-dispersive X-ray spectrometer (EDS) was engaged with BSE imaging to confirm the identity of any unhydrated or unreacted particles based on their elemental composition.

## Radiation shielding

As conducted in the preceding characterization tests and analyses, the radiation attenuation was evaluated for the control mix (CC), and those containing 10% and 15% GS.

### Concrete preparation

**Fig. 4:**
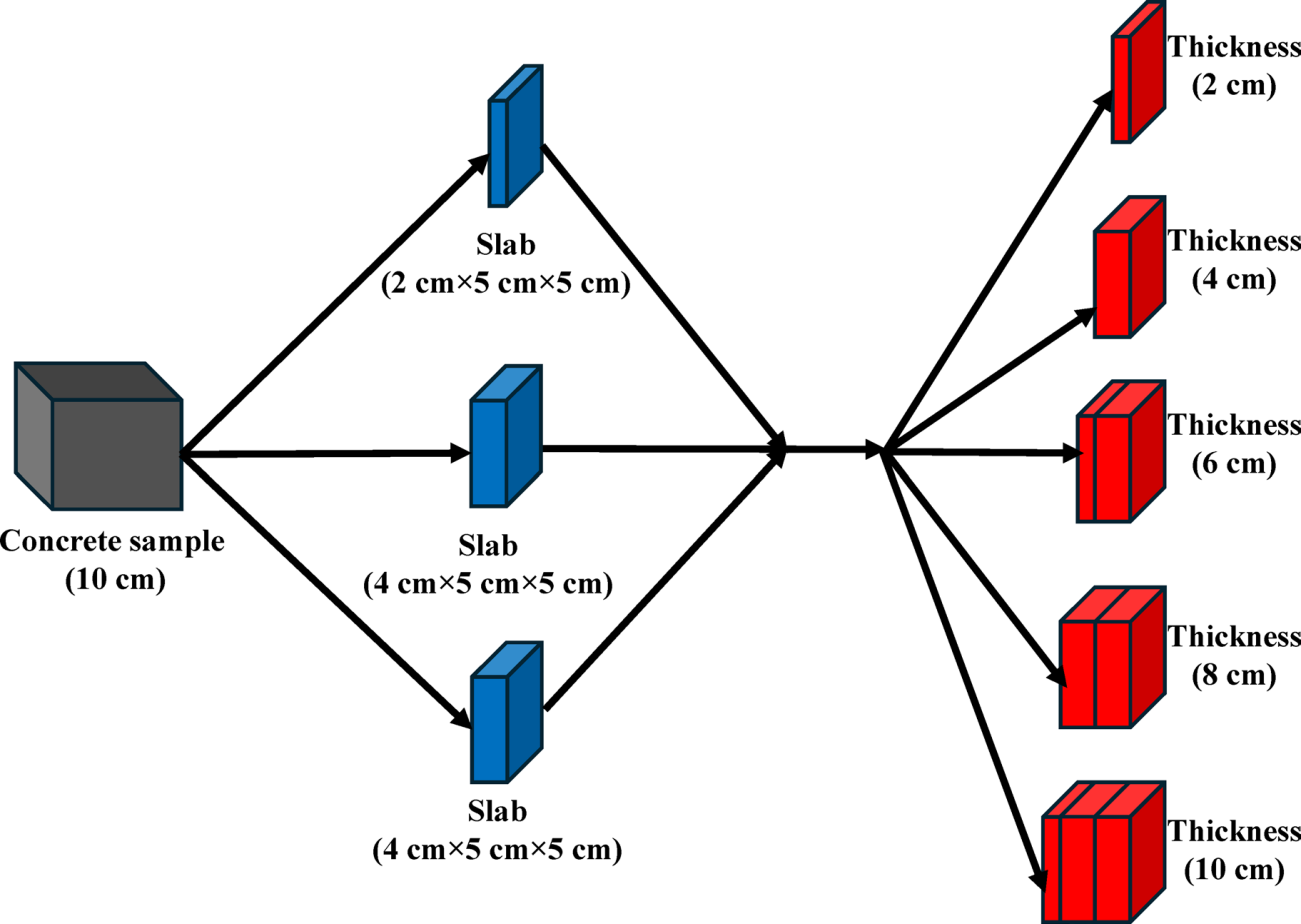
Schematic representation of the sectioning process used to prepare concrete samples for radiation shielding measurements.

Before implementing radiation shielding measurements, the concrete specimens were prepared by dividing them, as illustrated in Fig. 4. More specifically, following a 28-day curing age, a cubic concrete specimen of CC or GC (10 cm) was sectioned into three slabs of 5 × 5 cm with thicknesses of 2, 4, and 4 cm by a concrete disc saw at low speed with continuous cooling water. Moreover, the slabs were smoothed and levelled using SiC abrasive to remove any residual cutting debris and ensure a uniform surface. This was conducted to get them successively collected to attain total thicknesses of 2, 4, 6, 8, and 10 cm (Fig. 4).

### Radiation shielding measurements

**Fig. 5:**
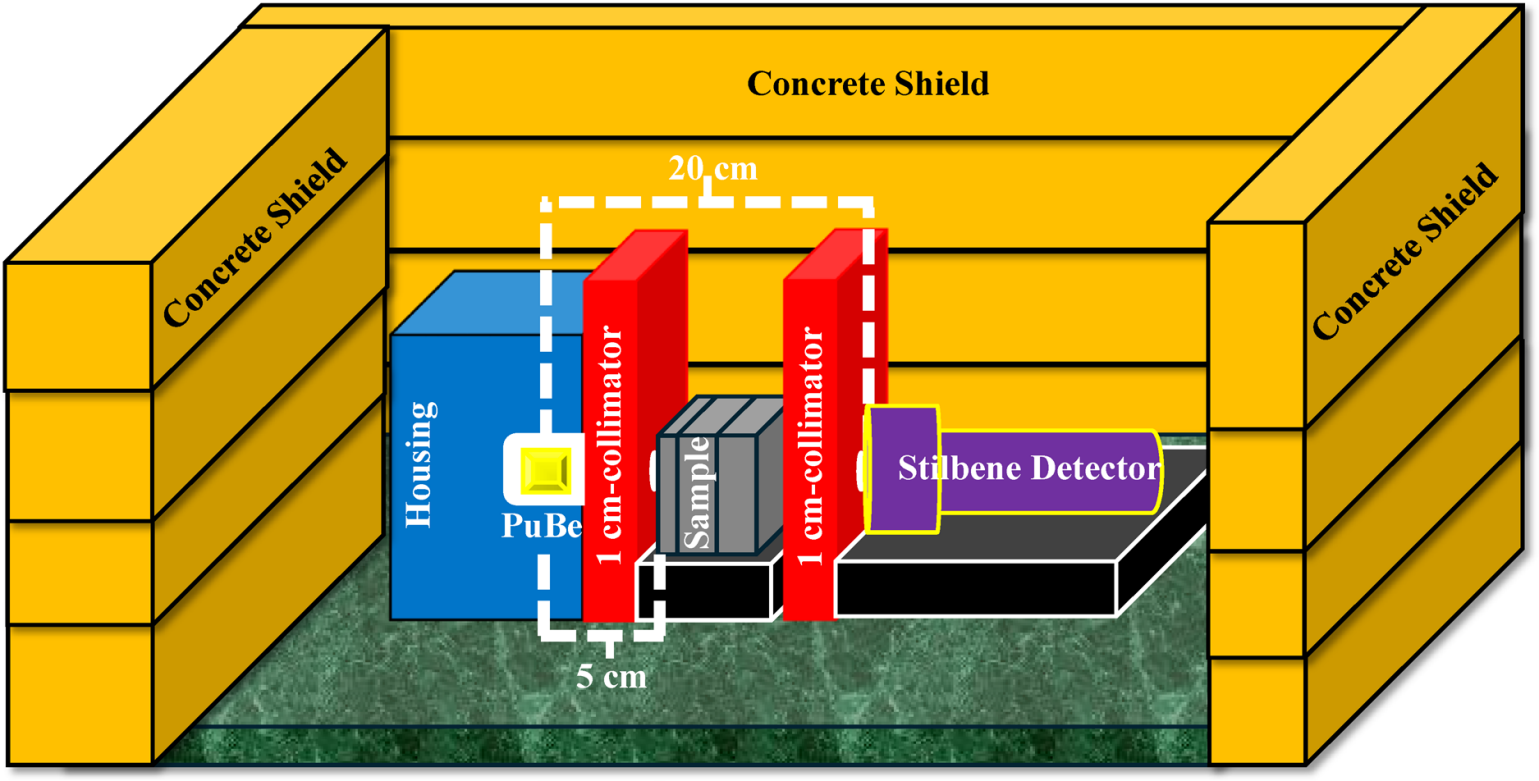
Design sketch showing experimental setup for measuring the radiation shielding of samples using a PuBe source and stilbene detector.

The radiation shielding experiments were executed at Nuclear Research Center, Egyptian Atomic Energy Authority. After a 28-days of curing, radiation attenuation characteristics of concrete specimens toward γ-rays and fast neutrons were systematically evaluated using a narrow beam transmission procedure. This configuration employed a collimated Pu–Be source complemented with a 1 cm single-face perforation (Fig. 5). Pu–Be source is recommended by ISO 8529–1 as a standard fast neutron source^68^, and it remains among the most utilized actinide/beryllium sources in neutron shielding investigations. Stilbene scintillator detector with a crystal dimension of 4 × 4 cm (Fig. 5) is appointed for measuring the transmitted fast neutrons and γ-rays. Like the Pu-Be source, collimation was conducted for the detector using Pb housing with a 1 cm slit to verify a narrow beam transmission and diminish the background radiation. The concrete slabs were located 5 cm away from PuBe, and detector was aligned coaxially with the source at a fixed distance of 20 cm and maintained at the same vertical level as the radiation source (Fig. 5).

An anticoincidence combined with a zero cross-over method was applied alongside pulse shape discrimination (PSD) to reliably separate recoil protons and electrons when neutrons and γ-rays interact with the detector, respectively^69^. This methodology allows a distinct neutron–gamma differentiation^70^. For detector calibration, PuBe, as well as ^137^Cs, and ^60^Co sources, were employed through their reference spectra of gamma-rays at 4.43 and 3.92 MeV, as well as 0.661 and 1.332 MeV, respectively. Within 0.80–11 MeV and 0.40–8.30 MeV, the attenuation performance of samples was evaluated for fast neutrons and γ-rays, respectively. For each concrete thickness, counting time was set over 300 s, with a total accumulation time of 1500 s per specimen. In parallel, the bare was measured over 300 s to keep statistical uncertainty at ± 4%. A digital counter was operated by a high voltage of −1900 V to counteract any possible energy irregularities in the radiation source. As shown elsewhere^71^, the experimental setup displays the electronic assembly of the fast neutron–gamma spectrometer, including dynode signal assembly from the photomultiplier tube. Besides, Table 2 presents the equations (Eqs. 1–5) applied to derive the essential parameters used in assessing the attenuation capability against fast neutrons and gamma-rays. The associated statistical uncertainties were determined under 4%, using Eqs. (6) and (7).

**Table 2:** Equations utilized to find shielding parameters for fast neutrons and γ-rays, including the corresponding expressions for uncertainty propagation.

No	Parameter	Symbol	Unit	Definition	Equation	Abbreviations
1	Effective macroscopic removal cross-section of fast neutrons	Σ_R_	cm^−1^	Possibility of fast neutron per unit path length to undergo a first interaction, removing it from group of uncollided fast neutrons	$N = N_{o} e^{{ - \Sigma_{R} {\kern 1pt} x}}$	N_0_ and N denote incident and transmitted fast neutrons intensities, respectively, *x*: specimen thickness (cm)
2	Linear attenuation coefficient of gamma-rays	μ		Fraction of shielded gamma-rays per thickness unit	$I = I_{0} e^{ - \mu x}$	I_0_ and I denote incident and transmitted γ-ray intensities, respectively
3	Mean free path	MFP	cm	Average distance between two successive collisions	$MFP = 1/_{R}$ $MFP = 1/\mu$	
4	Half value layer	HVL		Thickness decreasing incident radiation intensity to one-half	$HVL = \ln 2/_{R}$ $HVL = \ln 2/\mu$	
5	Tenth value layer	TVL		Thickness decreasing incident radiation intensity to one-tenth	$TVL = \ln 10/_{R}$ $TVL = \ln 10/\mu$	
6	Uncertainty propagation equations			$\Delta {\kern 1pt} (\mu ) = \frac{1}{x}\sqrt {\left( {\frac{{\Delta {\kern 1pt} I_{o} }}{{I_{o} }}} \right)^{2} + \left( {\frac{{\Delta {\kern 1pt} I}}{I}} \right)^{2} + \left( {\ln \frac{{I_{o} }}{I}} \right)^{2} \left[ {\left( {\frac{{\Delta {\kern 1pt} \rho }}{\rho }} \right)^{2} + \left( {\frac{{\Delta {\kern 1pt} x}}{x}} \right)^{2} } \right]}$	ρ: sample density	
7				$\Delta (\Sigma_{R} ) = \frac{1}{x}\sqrt {\left( {\frac{{\Delta {\kern 1pt} N_{o} }}{{N_{o} }}} \right)^{2} + \left( {\frac{{\Delta {\kern 1pt} N}}{N}} \right)^{2} + \left( {\ln \frac{{N_{o} }}{N}} \right)^{2} \left[ {\left( {\frac{{\Delta {\kern 1pt} \rho }}{\rho }} \right)^{2} + \left( {\frac{{\Delta {\kern 1pt} x}}{x}} \right)^{2} } \right]}$		

## Results and discussion

### Material characterization

Regarding the physical properties of studied materials, the bulk density of GS (1.16 ± 0.04 g/cm^3^) is lower than that of PC (1.60 ± 0.05 g/cm^3^). This difference in density can be assigned to a variance in the constituent minerals.

**Fig. 6:**
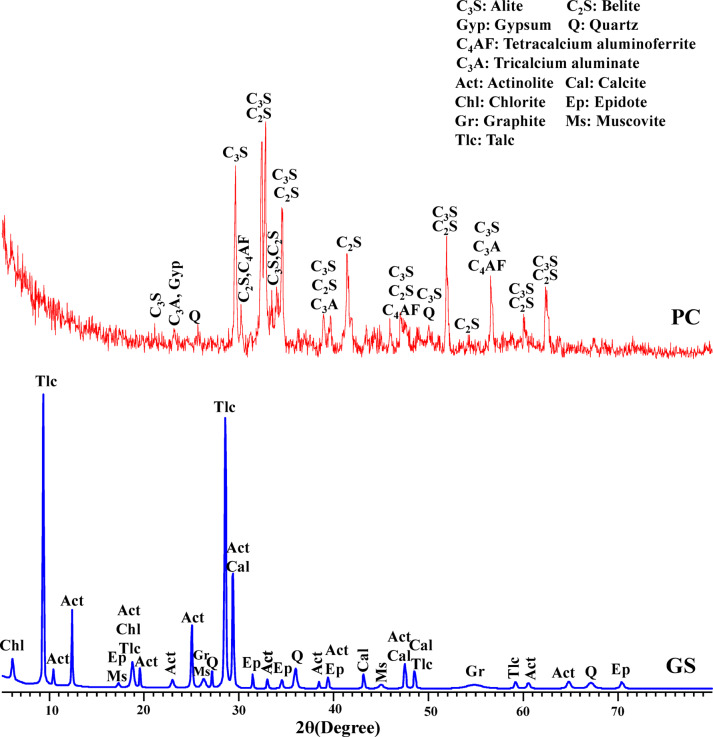
XRD patterns of PC and GS material.

As presented in Fig. 6, XRD analysis of PC exhibits the main phases represented in C_3_S, C_2_S, C_3_A, C_4_AF, and Gyp, which denote alite, belite, tricalcium aluminate, tetracalcium aluminoferrite, and gypsum, respectively, following ASTM C150^72^. Furthermore, a diffuse background in the diffraction pattern suggests the presence of amorphous phases, like glass materials. Otherwise, the XRD pattern of GS demonstrates that it is mainly composed of talc (Mg_3_Si_4_O_10_(OH)_2_) and actinolite (Ca_2_(Mg,Fe)Si8O_22_(OH)_2_), along with minor incidences of calcite (CaCO_3_), graphite (C), epidote (Ca_2_(Fe,Al)_3_(SiO_4_)_3_(OH), muscovite K_2_Al_4_[Si_6_Al_2_O_20_](OH,F)_4_, chlorite (Mg,Fe,Mn,Al)_12_[(Si,Al)_8_O_20_](OH)_16_, and quartz (SiO_2_). Among these, graphite is the least abundant mineral in the sample, followed by chlorite, epidote, muscovite, quartz, and actinolite, while talc is the most dominant phase. Therefore, the sample should be named as graphite-chorite-epidote-muscovite-quartz-actinolite-talc schist as recommended by the IUGS Subcommission, whereas the arrangement of minerals is in order of ascending modal abundance^73^. But it was abbreviated to graphite schist (GS) to simplify its name. More specifically, the main composing minerals of GS have lower densities; talc and actinolite. This reflects on the lower density of GS compared to PC.

As presented in Table 3, the chemical composition illustrates that GS contains significantly higher proportions of SiO_2_ (39.78%), MgO (27.38%), and LOI (16.38%) compared to PC, which has corresponding values of 19.42, 1.20, and 3.72, respectively. This can be attributed to the majority of talc (Mg_3_Si_4_O_10_(OH)_2_) and actinolite (Ca_2_(Mg,Fe)Si8O_22_(OH)_2_) in GS, as confirmed by the XRD results (Fig. 6). Otherwise, there is a superiority of CaO% in PC compared to GS due to enrichment of PC in calcium-bearing phases, particularly alite (2CaO·SiO_2_) and belite (2CaO·SiO_2_). In GS, the presence of CaO (11.85%) is largely due to the occurrence of calcite (CaCO_3_) and actinolite. Besides, the presence of Fe_2_O_3_ (4.19%) in GS can also be attributed to actinolite.

**Table 3:** Chemical composition of PC and GS identified by XRF.

Major oxides, wt. (%)	PC	GS
SiO_2_	19.42	39.78
Al_2_O_3_	4.72	0.27
Fe_2_O_3_	4.85	4.19
CaO	62.72	11.85
MgO	1.20	27.38
Na_2_O	0.42	0.01
K_2_O	0.16	0.01
TiO_2_	–	0.06
P_2_O_5_	–	0.01
SO_3_	2.65	0.01
Cl	0.09	0.01
LOI	3.72	16.38

**Fig. 7:**
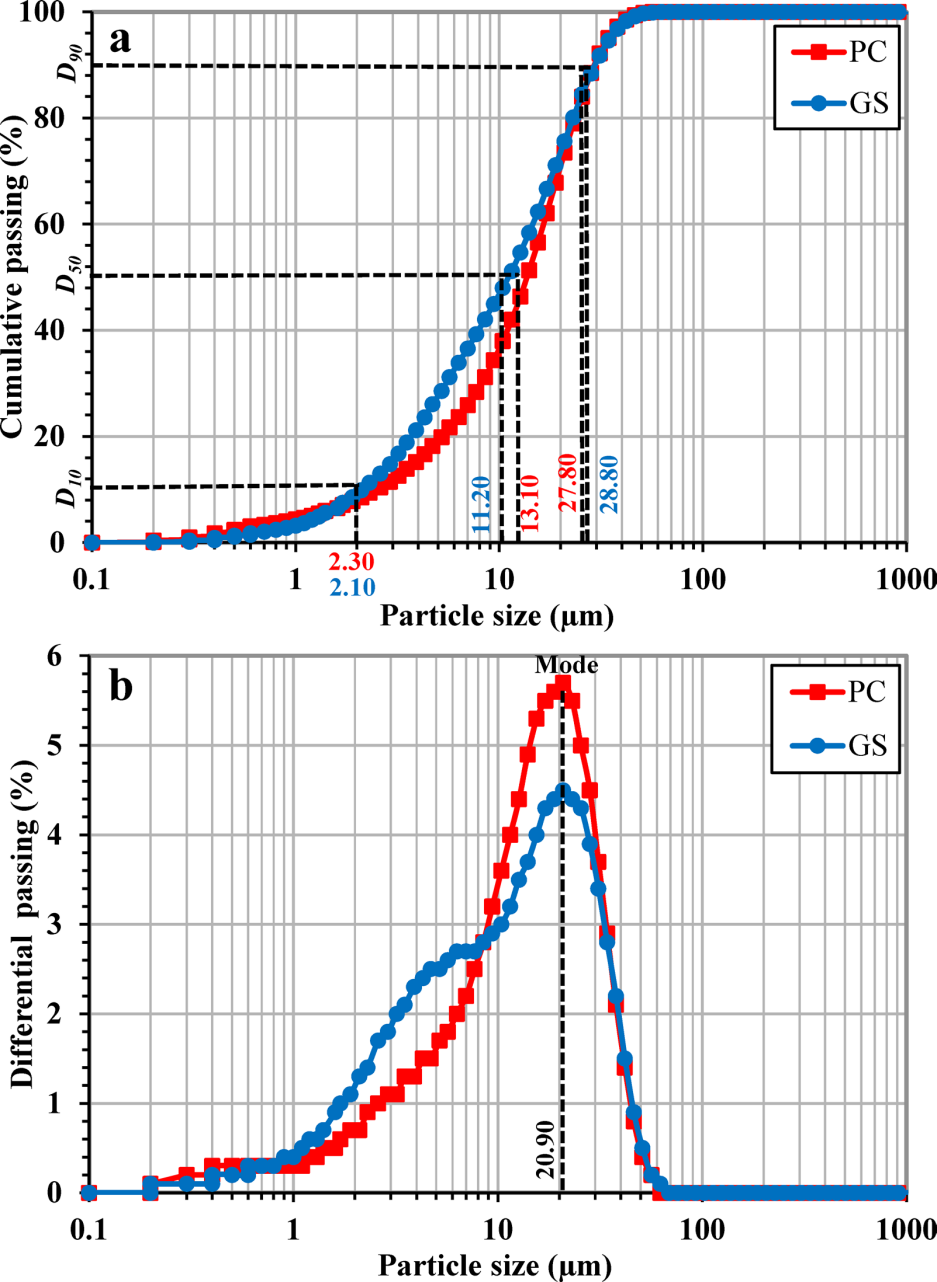
Particle size distribution (PSD) of PC and GS: (**a**) Cumulative PSD and (**b**) Differential PSD.

Figure 7 illustrates the particle size distribution (PSD) of PC and GS samples, plotted as cumulative PSD (Fig. 7a) and differential PSD (Fig. 7b) curves. Both materials exhibit highly comparable PSD profiles, reflecting the effectiveness of the applied grinding methodology for GS. This similarity is further confirmed by the close agreement in the characteristic particle size parameters (*D*_10_, *D*_50_, and *D*_90_), as well as the dominant modal particle size. The *D*_10_, *D*_50_, and *D*_90_ values were derived from Fig. 7a, while the mode was obtained from Fig. 7b. Specifically, GS exhibits slightly lower *D*_10_ and *D*_50_ values of 2.10 and 11.20 µm, compared to 2.30 and 13.10 for PC, respectively. Moreover, both samples exhibit comparable *D*_90_ values of 27.80 µm and 28.80 µm, respectively (Fig. 7a). Figure 7b displays a dominant peak at approximately 20.90 µm for both samples, corresponding to the primary modal particle size. Overall, these results indicate that GS particles are distributed within a particle size range comparable to that of PC, with a slight shift toward finer sizes as reflected by the lower *D*_10_ and *D*_50_ values. This is particularly important for ensuring comparable packing density and potential reactivity and filling effect of GS^74^. Moreover, the similarity in PSD contributes to a more uniform particle size distribution, which in turn promotes better homogeneity during the dry mixing^75^.

BET surface area analysis reveals that the GS exhibits a two-fold higher BET surface area (35.25 m^2^/g) compared to PC (16.81 m^2^/g), verifying the previous findings of particle size analysis. Moreover, this refers to the possibility of increased nucleation spots for hydration phases and boosted pozzolanic reactivity^76^. Also, this can reflect on the filling effect of GS^74^.

**Fig. 8:**
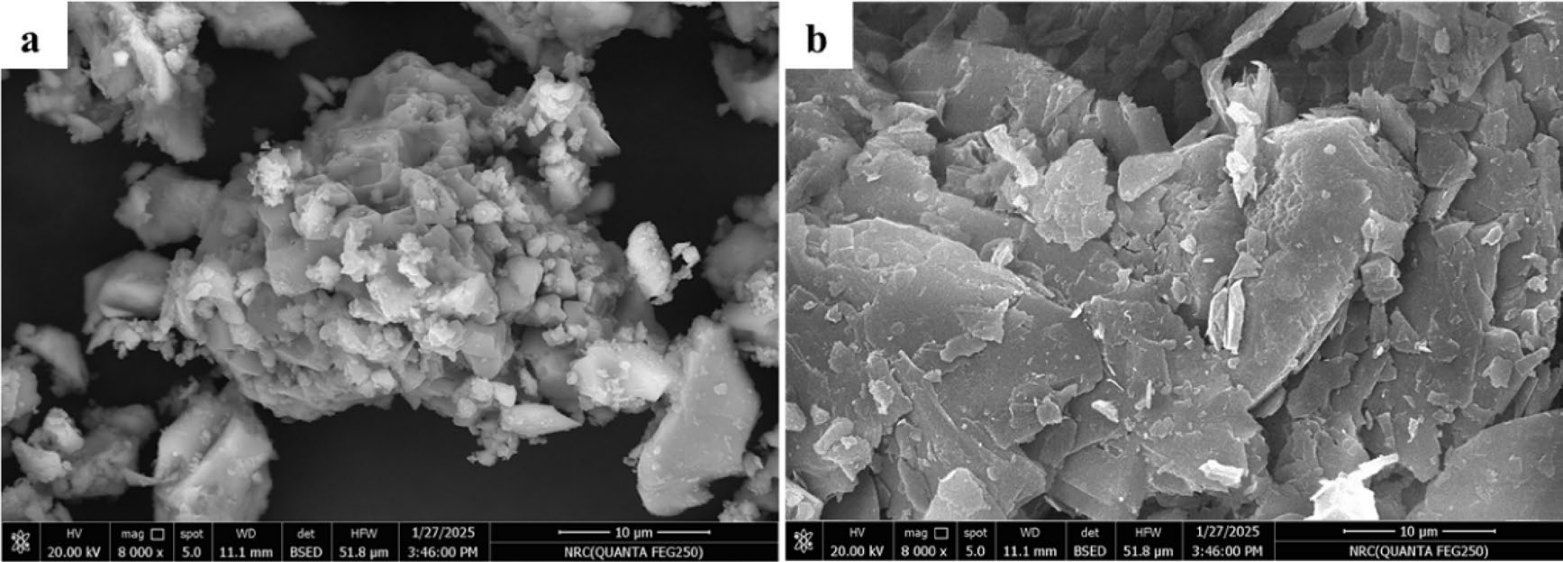
SEM images of (**a**) PC and (**b**) GS.

As demonstrated in SEM images, there is a variation in the morphological properties of PC and GS particles (Fig. 8). Figure 8a demonstrates that the PC particles are angular and rough, with sharp edges and a bumpy surface morphology. Otherwise, Fig. 8b illustrates that GS particles are like agglomerated smooth platelets with a layered texture and relatively large surface area. This morphology can be related to the nature of smooth, flaky talc and sub-columnar actinolite minerals, as confirmed by XRD analysis (Fig. 6). On the other side, the smooth surfaces of GS particles limit their ability to retain mixing and hydration water^77^, leading to an adverse impact on hydration rate.

**Fig. 9:**
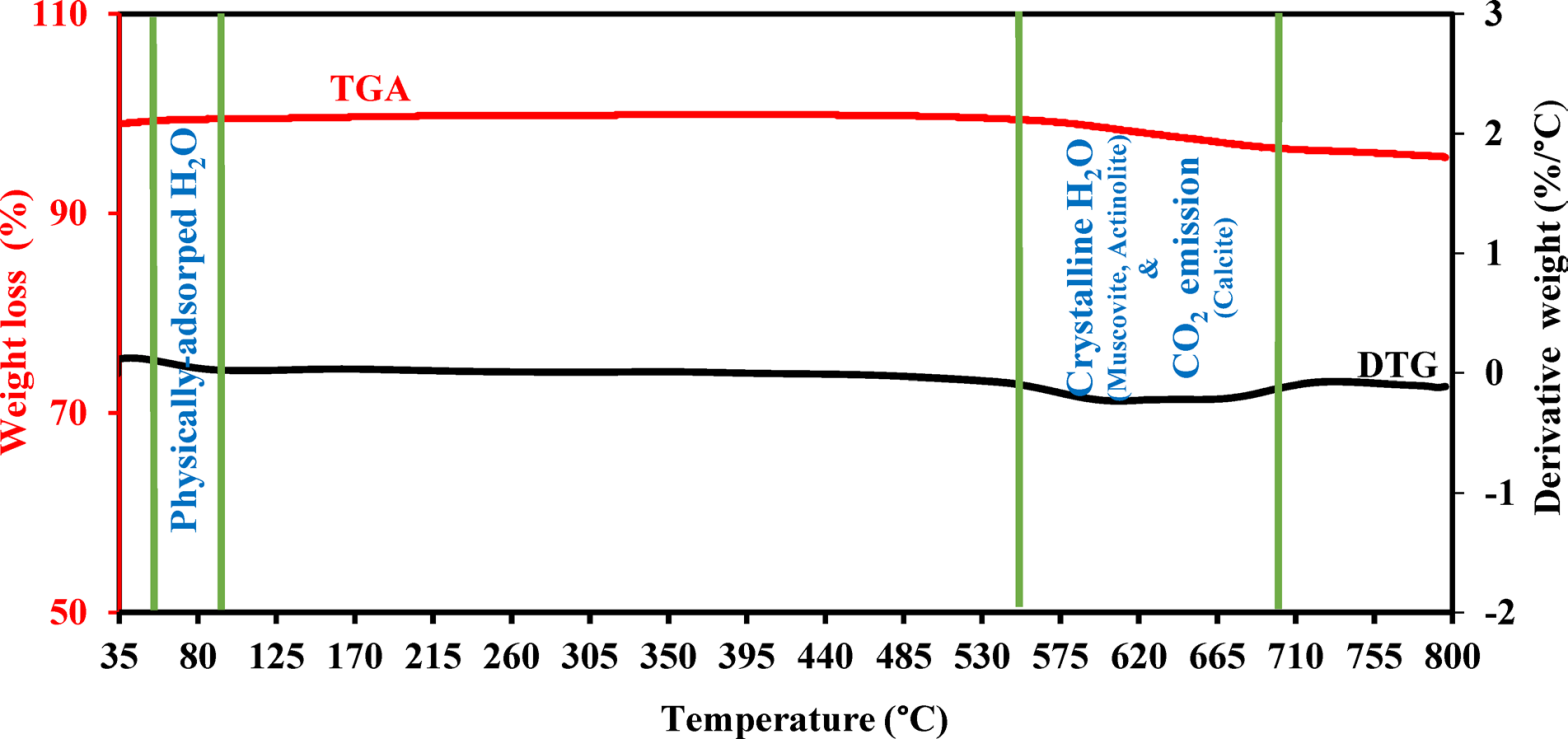
TGA and DTG curves of GS material.

As shown in Fig. 9, the initial weight loss observed at about 50–90 °C can be assigned to the evaporation of physically adsorbed water. Otherwise, the minor decompositions occurring between 550 and 700 °C likely correspond to release of crystalline water from the mineral constituents (i.e., muscovite and actinolite)^78,79^. Also, this temperature range can be assigned to the CO_2_ emission from calcite decomposition^80,81^. Beyond 700 °C, the GS sample reveals stable thermal behaviour up to 800 °C. Therefore, it can be deduced that GS material reveals thermal stability up to 800 °C, with only minor decompositions within 550–700 °C range. Consequently, GS can serve as a thermally stable SCM, having promising benefits for fire resistance applications. This thermal behaviour may also reflect improved fire performance in cement^82^. Consequently, it efficiently optimizes the thermal durability of concrete mixtures that incorporate GS, assuming that it meets the appropriate requirements^83^.

**Fig. 10:**
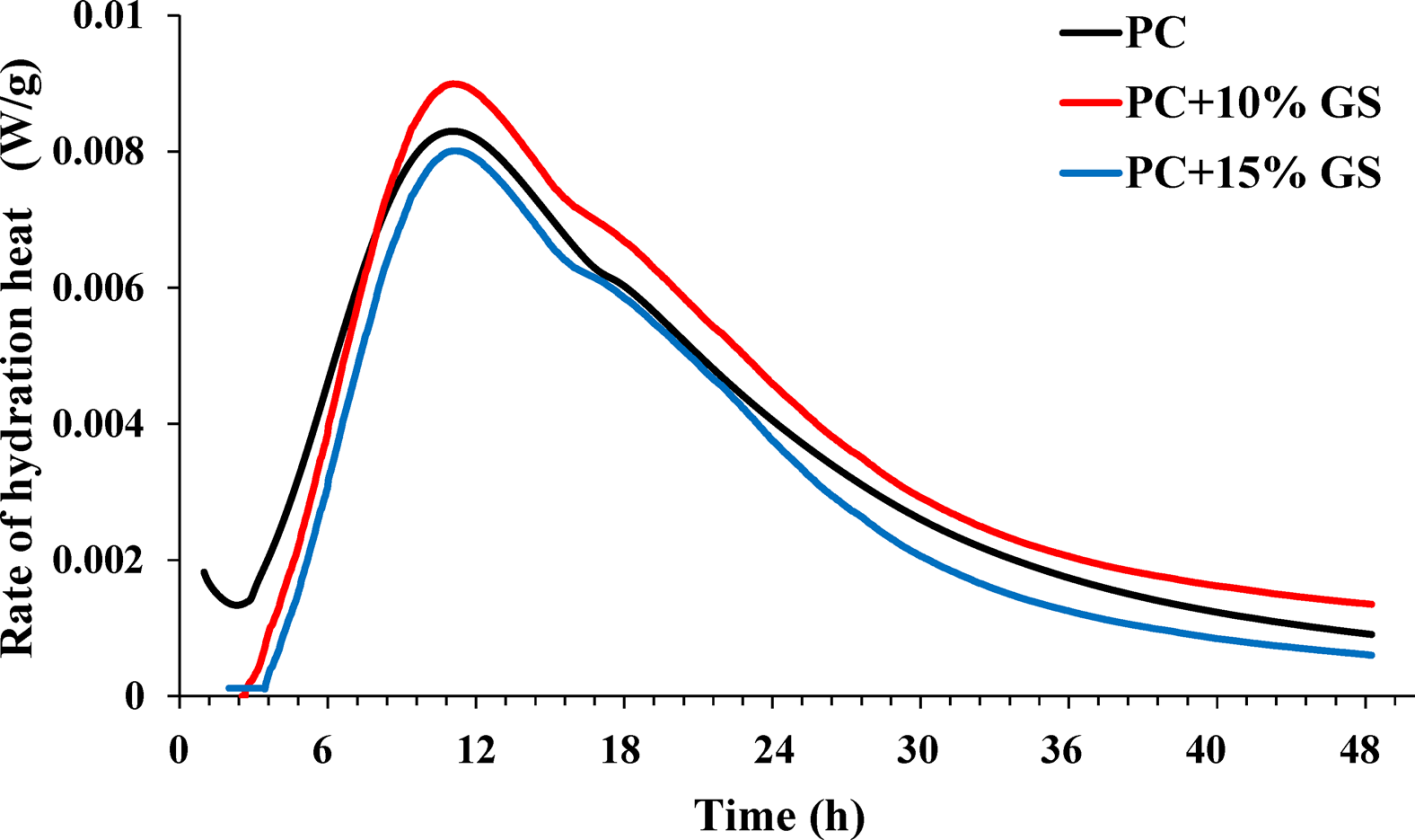
Rate of hydration heat evolution for cementitious materials composed of PC (reference) and PC substituted with 10% and 15% GS.

**Fig. 11:**
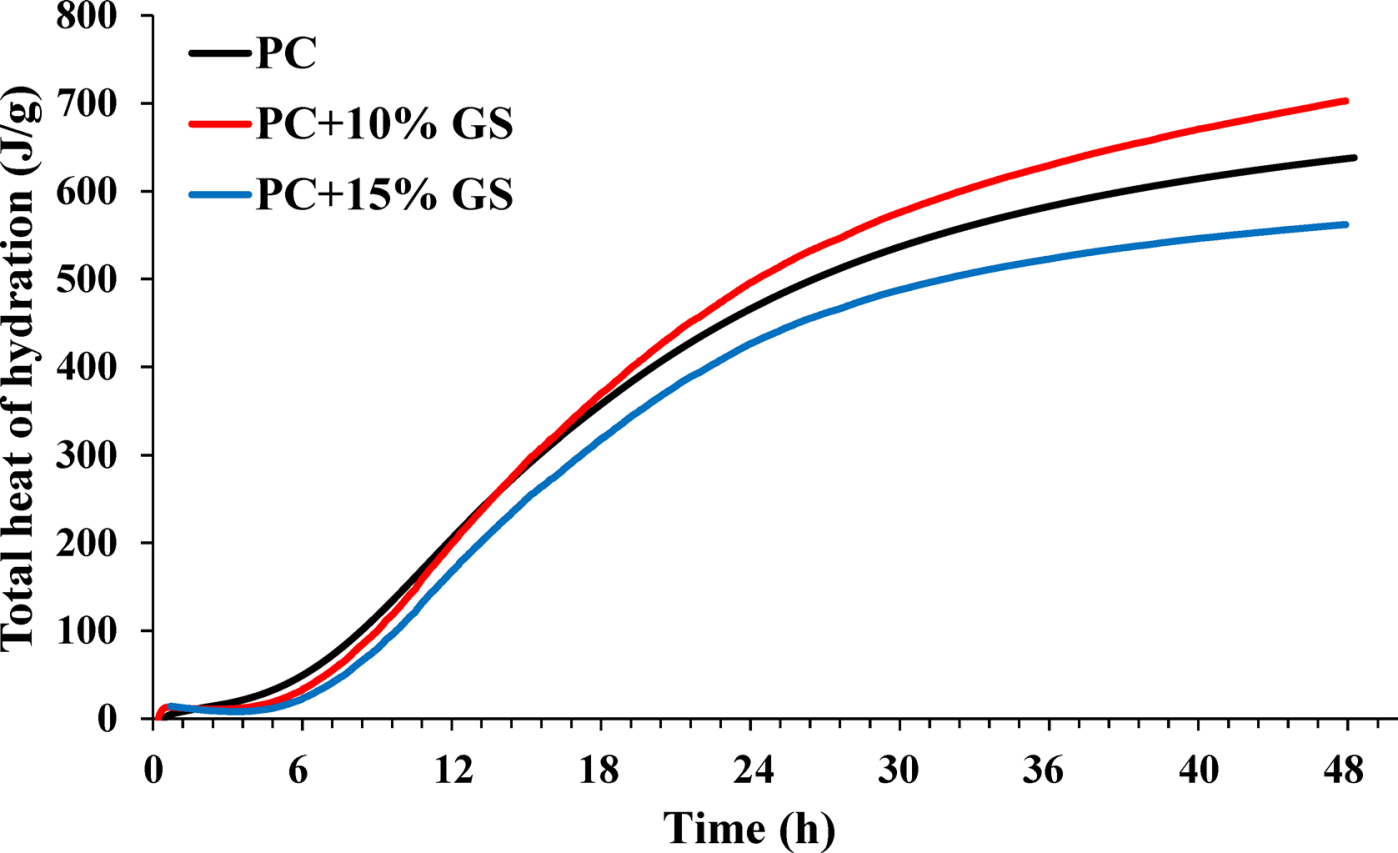
Cumulative heat release during hydration for cementitious materials composed of PC substituted with 10% and 15% GS.

Hydration heat evolution and cumulative heat release of PC (reference), PC + 10% GS, and PC + 15% GS reveal distinct differences in reaction kinetics due to GS incorporation, as shown in Figs. 10 and 11. It was observed that PC mix shows the highest rate of hydration heat (Fig. 10) and the greatest cumulative heat release (Fig. 11) during the first ~ 10 h of hydration, reflecting the rapid early hydration of clinker phases and the higher availability of reactive constituents. This behavior is consistent with the higher early heat flow observed for PC and supposes its superior early-age strength development. In contrast, during this early hydration period, both PC + 10% GS and PC + 15% GS display lower hydration heat rates and reduced cumulative heat release, with a more pronounced and consistent reduction for PC + 15% GS. This reduction is mainly attributed to clinker dilution, which limits the extent of early hydration reactions. Conversely, beyond ~ 10 h, a distinct change in the hydration behavior is observed for PC + 10% GS, where its rate of hydration heat and cumulative heat release surpass those of PC (Figs. 10, 11). This suggests a more sustained hydration process in PC + 10% GS, which can be attributed primarily to physical filler and nucleation effects rather than enhanced early reactivity. More specifically, the presence of finely dispersed GS particles supplies additional nucleation sites for hydration products due to its higher BET surface area (35.25 m^2^/g) compared to PC (16.81 m^2^/g). Consequently, this promotes continued hydration of the remaining clinker phases at later stages, leading to a gradual increase in rate of hydration heat and high cumulative heat release (Figs. 10, 11). In contrast, the PC + 15% GS mixture keeps a significantly lower rate of hydration heat and reduced cumulative heat release throughout the hydration period, indicating the dilution effect domination and hydration progress limitation due to the reduced availability of reactive clinker phases.

**Fig. 12:**
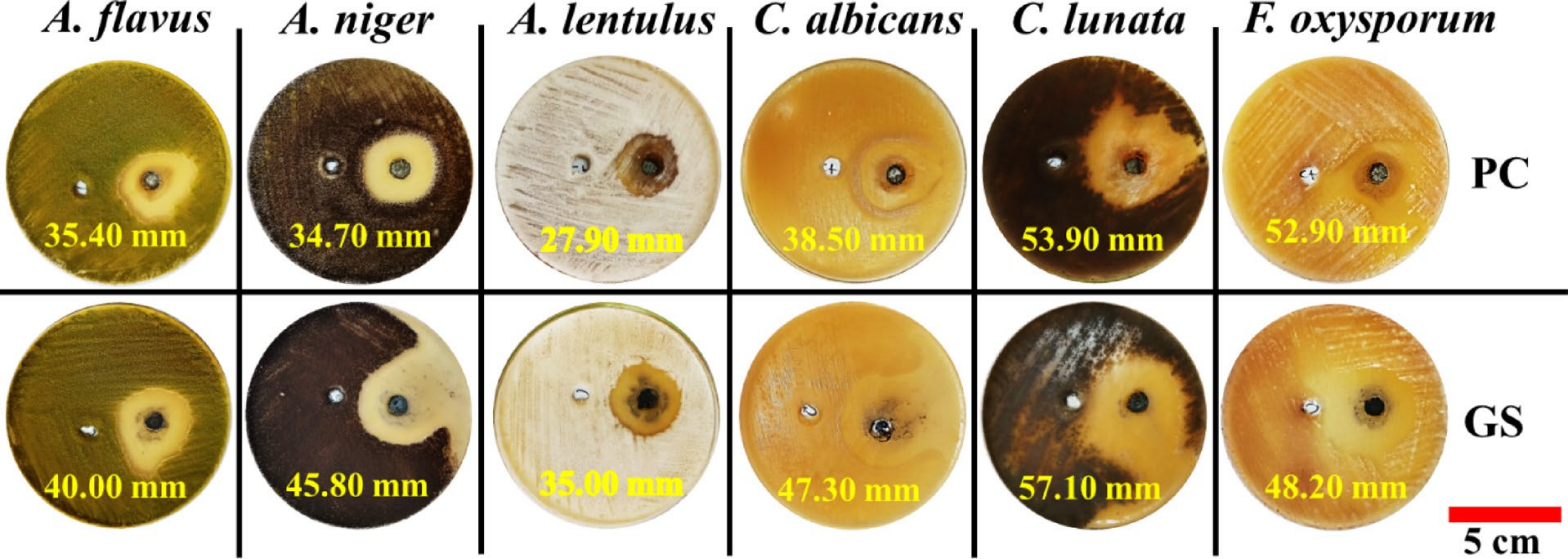
Photographic images of antifungal activity in PC and GS when exposed to multiple fungal strains.

Regarding antifungal activity, Fig. 12 illustrates that both PC and GS possess a type of antifungal activity as indicated by the inhibition zones (mm) on each disc. Relative to PC, GS has almost superior inhibitory impacts against all examined fungal strains, ranging from about 35.00 to 57.10 mm: filamentous and yeast types (i.e., *A. flavus*, *A. niger*, *A. lentulus)*, *C. albicans*, *C. lunata*, and *F. oxysporum*). Only comparable inhibitory abilities are observed against *F. Oxysporum* for both PC and GS (52.90 and 48.20 mm, respectively). This superior performance of GS implies the presence of significantly higher concentrations of active elements that can hinder fungal growth with respect to PC. Commonly, antifungal activity of materials can arise due to many features, with surface area being particularly critical. A higher surface area has a profound impact on the elemental composition, chemical reactivity, and pH modification of antifungal materials^84^.

Explicitly, the higher BET surface area of GS (35.25 m^2^/g) substantially improves the chemical reactivity of embedded heavy metals, particularly iron (Fe), which is known for malignant effects on fungal cells (Table 3). More specifically, the redox cycling between ferric and ferrous forms leads to formation of reactive oxygen species, which in turn damage fungal cells^85,86^.

Although magnesium (Mg) is not categorized as a toxic heavy metal, its oxide (MgO) has been reported to disrupt fungal membranes, inhibit spore germination, and induce oxidative stress^87^. This can be due to the morphological modification of fungal cells, suppressing the mycelial growth, and inhibition of sporulation as a result of the direct reactions between MgO and fungal cells^88^. Moreover, the high MgO content in GS, combined with its smaller particle size (*D*_50_ = 11.20 µm), compared to that of PC (*D*_50_ = 13.10 µm), contributes to oxidative stress and subsequent cell damage^89^. In the same sense, the impact of MgO is augmented through the high surface of GS (35.25 m^2^/g) that enhances MgO chemical reactivity. Interestingly, these findings indicate that GS is a promising SCM, which can improve the antimicrobial efficacy of PC when used as a partial replacement.

### Concrete characterization

#### Workability and physico-mechanical characterization

As exhibited in Table 4, slump test results of fresh concrete display that GS incorporation slightly diminishes concrete workability. In particular, the increase in GS replacement level results in comparable slump values of GC10 and GC15 compared to CC. This could be assigned to high BET surface area of GS material (35.25 m^2^/g), resulting in demanding more water and then a reduction in slump values^90,91^.

**Table 4:** Slump values of fresh concrete mixtures.

Concrete type	Slump (mm)
CC	75
GC10	70
GC15	65

As listed in Table 5, it was found that concrete specimens containing GS (i.e., GC10 and GC15) have lower densities compared to that of the CC specimen. Explicitly, the densities of GC10 and GC15 are slightly lower than that of CC. This can be assigned to the lower density of GS material (1.16 ± 0.04 g/cm^3^) than PC (1.60 ± 0.05 g/cm^3^). Unlike density, the water absorptions and porosities of GC10 and GC15 are higher than those of CC. This could be assigned to effect of high content of talc as hydrophobic mineral in GS material^92^.

**Table 5:** Physical properties of concrete.

Concrete type	Density (g/cm^3^)	Water absorption (%)	Porosity (%)
CC	2.30	1.51	3.52
GC10	2.25	1.71	3.94
GC15	2.20	1.82	4.05

**Fig. 13:**
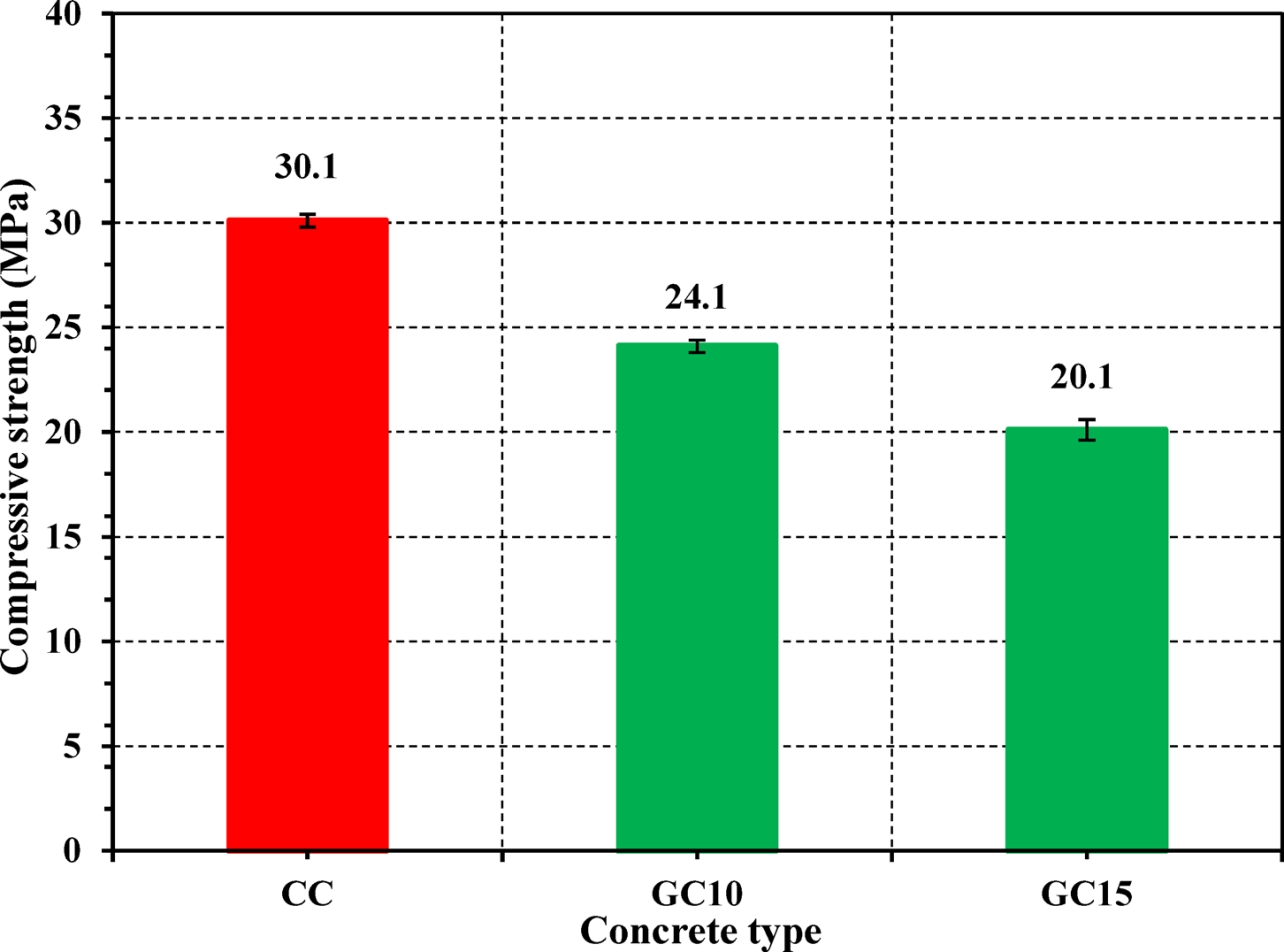
Compressive strength of concrete specimens after 28 days of curing for CC, GC10, and GC15.

As revealed in Fig. 13, the compressive strength (*f*_*c*_) results of CC, GC10, and GC15 demonstrate that the GC10 specimens, with the most effective replacement level, achieve minimal adverse effects on *f*_*c*_ compared to CC specimen after 28 days of curing. Therefore, GC10 is chosen as the preferred concrete for further testing, including residual mechanical, hydration, and radiation shielding assessments.

**Fig. 14:**
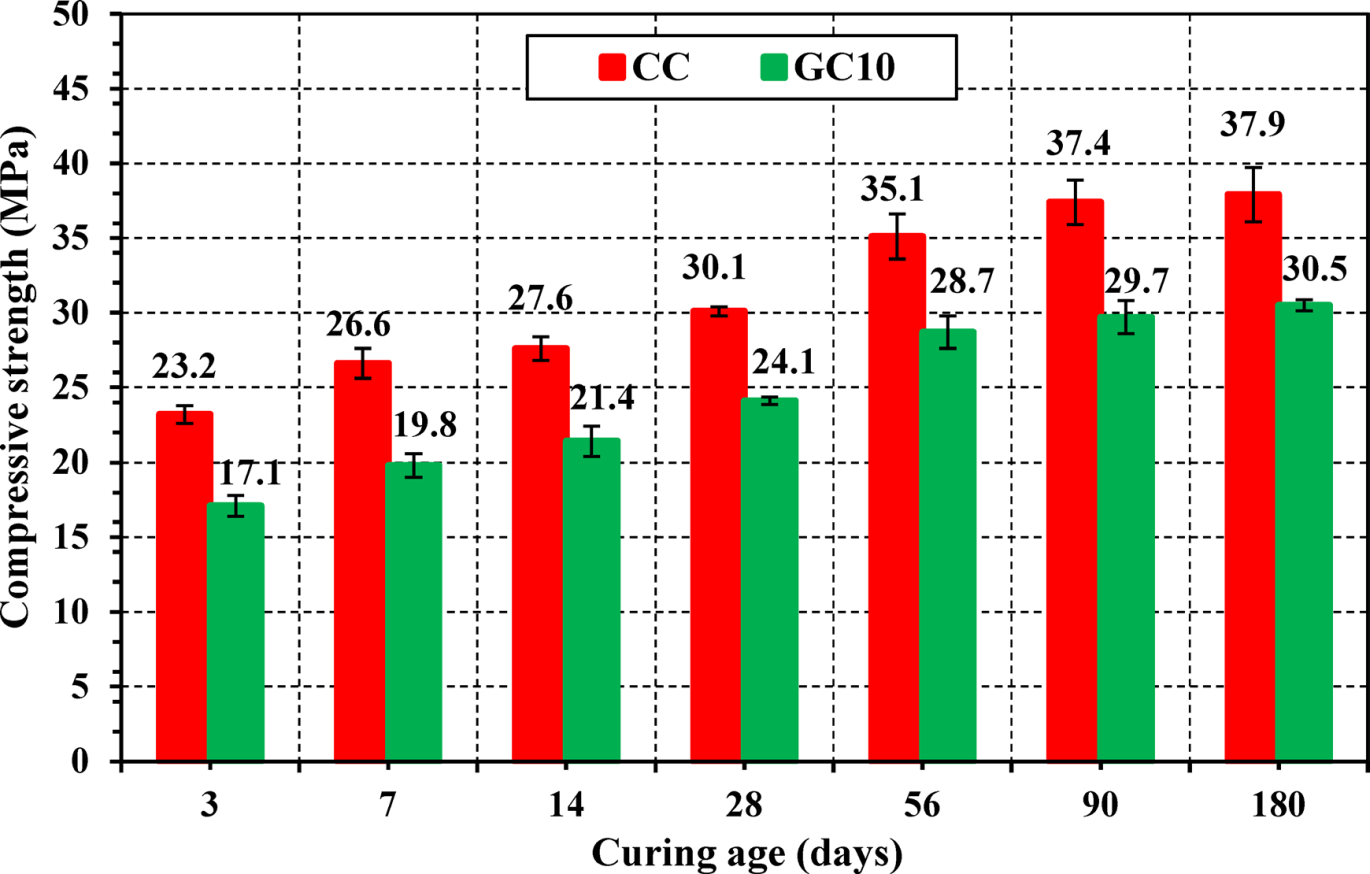
Compressive strength of CC and GC10 specimens at different ages.

Figure 14 exhibits the relationship between *f*_*c*_ of CC and GC10 over various curing days (3, 7, 14, 28, 56, 90, and 180 days). GC10 shows lower *f*_*c*_ values compared to CC over all curing periods, indicating the detrimental effect of 10% GS substitution. However, this deleterious influence decreases with increasing curing age, suggesting continued hydration and long-term strength development up to 180 days for all specimens. Explicitly, the compressive strength difference between CC and GC10 decreases from 35.70% at 3 days to 24.30% at 180 days. This is consistent with the findings of other filling materials^93^. Additionally, the compressive strength of GC10 improves by 78% from 3 to 180 days, compared to a 63% increase for CC. This is consistent with the findings of isothermal calorimetry.

The observed trend can be ascribed to progressive deposition of hydration products over curing days, with a specific increase in the later ages. This infers that GS contribution to strength development is more likely related to enhanced particle packing and matrix densification rather than secondary hydration reactions (i.e., pozzolanic reactions)^94^. Also, the high surface area of GS can provide an extra appropriate nucleation substrate for growing the hydration products over the curing age^95^. Consequently, GS could have a higher accelerating influence on the hydration process^96^. However, the improving effect of GS demonstrates a prolonged and sustained influence on *f*_*c*_. This outcome may be assigned to the relatively high w/b of 0.50 used in the recent work. The recent w/b can deteriorate the particle packing density and then the strength and durability^97,98^. Therefore, it is recommended to experiment with 10% GS, having lower w/b in future research.

#### Concrete hydration and microstructure characterization

**Fig. 15:**
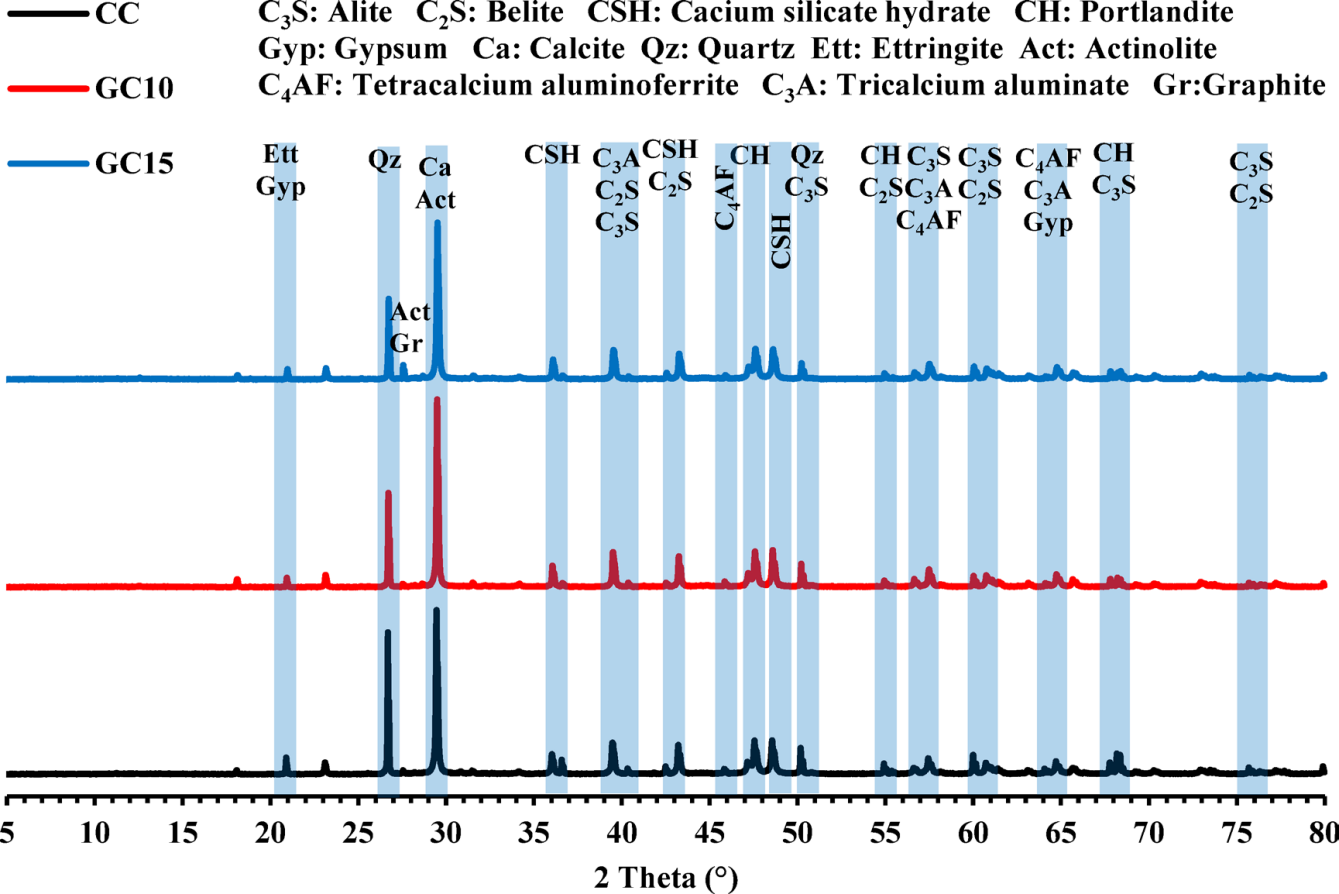
XRD patterns of concrete specimens (CC, GC10, and GC15) after 28 days of curing.

As presented in XRD results (Fig. 15), the yield of hydration products in the CC specimen differs from that in the GC10 and GC15 specimens. These differences are evident in the peak positions and intensities of ettringite (Ett), portlandite (CH), and calcium silicate hydrate (CSH). Moreover, their unreacted cement phases, considering alite (C_3_S), belite (C_2_S), gypsum (Gyp), tricalcium aluminate (C_3_A), and tetracalcium aluminoferrite (C_4_AF) have different intensities. Explicitly, the peaks of Ett, CSH, and CH in GC10 and GC15 have lower intensities compared to CC. Moreover, the unreacted cement phases in GC10 and GC15 follow the same reducing trend as in the hydration products compared to CC. These reducing trends of both hydration products and cement phases are more prominent in GC15 than those in GC10. This can be ascribed to the higher substitution level of cement with GS. Also, the malignant influence of GS on cement hydration indicates that GS is not a pozzolanic material. Furthermore, the deleterious effect of GS can be due to their constituting minerals especially talc mineral that creates an insulating film around cement particles, preventing the hydration process^99,100^. Moreover, the smooth surfaces of minerals such as actinolite fibers are not preferable for SCMs due to their low retention of the mixing and hydration water^77,100^. Otherwise, the increase in the replacing ratio of PC by GS results in the presence of graphite and actinolite peaks, as well as augments of calcite, as shown in GC15 (Fig. 15).

**Fig. 16:**
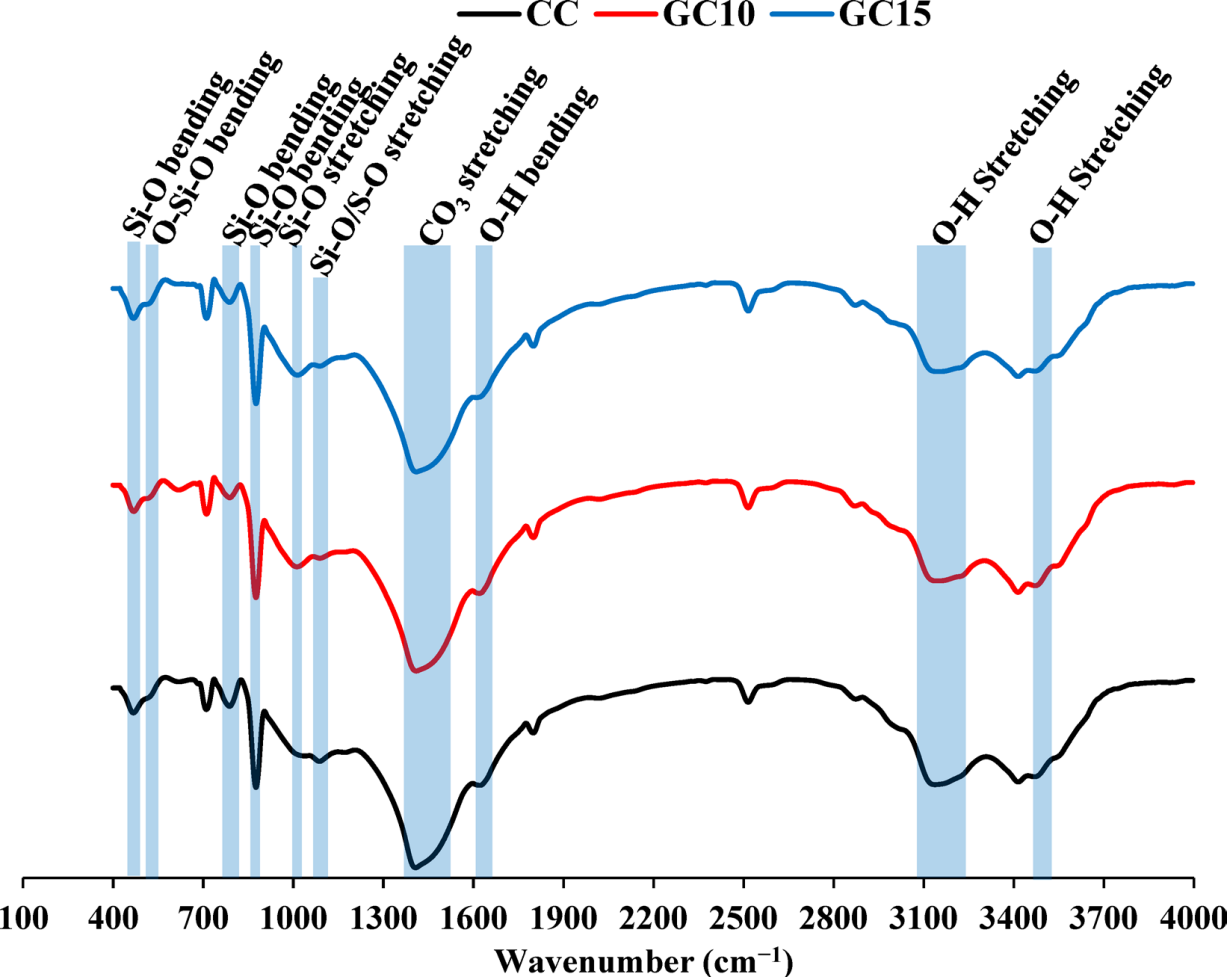
FTIR spectra of concrete specimens (CC, GC10, and GC15) after 28 days of curing.

Figure 16 shows the FTIR spectra with functional groups of CC, GC10, and GC15. These groups show noticeable variations in intensities or slight shifts in frequencies with GS addition. The bands at 460–475 cm^−1^ and 520–525 cm^−1^ are overlapping silicates related to Si–O and O–Si–O bending vibrations, which could indicate silicate phases of the cement or concrete. These bands reveal a slight increase in intensity in GC10 and GC15 compared to CC. This suggests a contribution of siliceous phases, specifically actinolite mineral incorporated with GS addition^101^. The bands located at 790–800 and 1100 cm^−1^ are related to Si–O bending and stretching vibrations, respectively, which are attributed to CSH^102,103^. A conspicuous decline is observed in the intensity of these bands in GC10 and GC15 compared to CC due to the clinker dilution caused by partial PC replacement with GS. The bands appearing at 870–880 cm^−1^ and 1400–1450 cm^−1^ are correlated to Si−O bending and CO_3_^−2^ stretching^5^ associated with sand and dolomitic limestone aggregates, respectively. As the aggregate content remains constant, these bands show insignificant intensity variation among the different mixtures. There is a new appearance of bands at 1012–1020 cm^−1^ in GC10 and GC15 compared to CC. These bands are assigned to Si–O stretching vibrations, which could be assigned to talc and actinolite minerals due to the increasing substitution of PC by GS^101,104^. Otherwise, the band at 1100 cm^−1^ is related to S–O stretching vibrations of sulphate phases^105,106^, showing the lowest intensity in GC15. Moreover, the bands at 1640–1650 cm^−1^ are related to O–H bending vibrations of sulphate phases^107^, which progressively decrease from CC to GC10, and GC15, respectively. These sulphate groups are commonly associated with the decline in ettringite or gypsum owing to the reduction in hydration products or substituted PC amount. Furthermore, the O–H stretching illustrated at 3120–3130 and 3450–3550 cm^−1^ can be assigned to the presence of CH^108,109^. The intensities of these bands are lower in GC10 and GC15 compared to CC, supporting the reduced hydration extent with increasing GS substitution. These findings are correlated with those previous ones of XRD (Fig. 15).

**Fig. 17:**
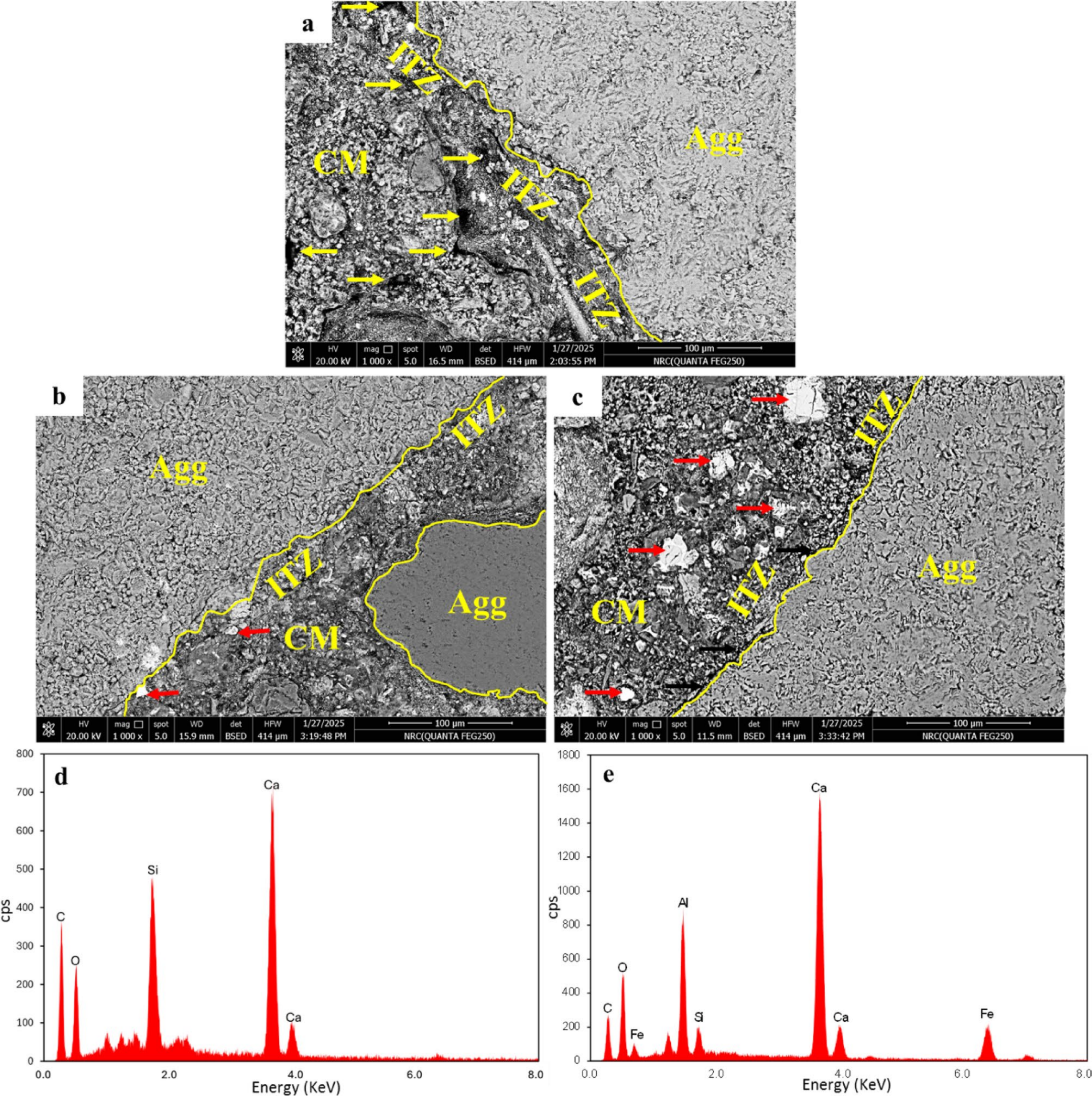
SEM-BSE images showing effect of GS replacement levels on the microstructural features of concrete: (**a**) CC, (**b**) GC10, (**c**) GC15, and (**d**, **e**) elemental composition of unreacted PC particles by EDS.

Regarding the influence of GS on the microstructural properties of concrete samples, SEM-BSE images (Fig. 17) were captured for CC (control), GC10 (the more effective replacement level), and GC15 (with the highest GS ratio) at the same magnification of 1000x. This allowed for a comparative analysis of the microstructural features, including pore structure distribution and the presence of unhydrated particles. Compared to CC (Fig. 17a), the 10% GS (GC10) and 15% GS (GC15) promote some ameliorations in the microstructure of the cement matrix, with the most noticeable effect seen in GC10 (Fig. 17b). This can be elucidated from the improved pore structure of GC10 and GC15 (Fig. 17b, c) due to the filling effect of GS material with lesser particle size (*D*_50_ = 11.20 µm) compared to PC (*D*_50_ = 13.10 µm)^74^. However, this enhancing effect was not operative due to the prevailing white particles, representing unreacted cement remnants with intact cores (indicated by red arrows), which are clearly visible in the cementitious matrix of both GC10 and GC15, with a more noticeable occurrence in the latter (Fig. 17). These particles were verified by the elemental composition acquired by EDS (Fig. 17d, e). Moreover, this observation is consistent with further high-water absorptions and porosities of GC10 and GC15 compared to those of CC (Table 5). Consequently, this indicates that the GS contribution to microstructure development could depend on the particle packing and densification rather than secondary hydration reactions (i.e., pozzolanic reactions)^94^.

Simultaneously, the region near the aggregate (Agg), known as ITZ, shows further improvement in GC10 compared to CC (Fig. 17a, b). This can be demonstrated from the particle packing, improved pore structure, and the absence of microcracks. However, the enhancing impact of these features can be not well active or effective due to some unhydrated cement particles observed in ITZ, as indicated by red arrows (Fig. 17b). This confirms the filling effect of GS rather than the pozzolanic impact as previously mentioned. This improvement also correlates with the modest improvement of compressive strength observed in GC10. On the other hand, in GC15, in addition to existence of unhydrated cement particles in cementitious matrix (marked by red arrows), microcracks were observed in the ITZ, as depicted by black arrows in Fig. 17c. This highlights the robust detrimental role of 15% GS on the microstructure and then the concrete strength.

### Radiation shielding

**Fig. 18:**
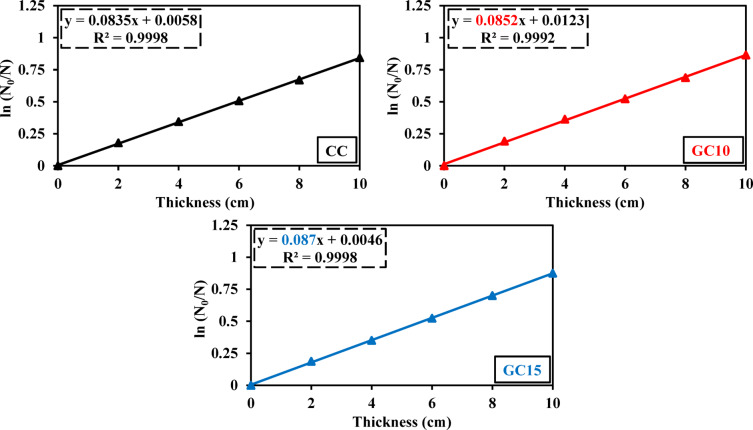
Variation of ln (N_0_/N) with thickness for CC, GC10, and GC15 specimens.

**Fig. 19:**
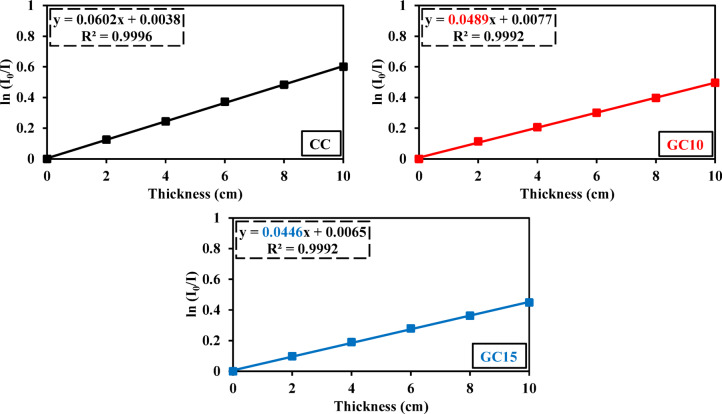
Variation of ln (I_0_/I) with thickness for CC, GC10, and GC15 specimens.

Figures 18 and 19 show linear relationships between the sample thickness (cm) as well as ln (N_0_/N) and ln (I_0_/I), respectively, for concrete mixes of CC, GC10, and GC15, in agreement with the Beer–Lambert law. From the straight-line slopes in Figs. 18 and 19, Σ_R_ (cm^−1^) and µ (cm^−1^) for each specimen are determined for each sample, with R^2^ values > 0.99, confirming an excellent linear correlation (Figs. 18, 19 and Tables 6, 7). Based on these obtained values of Σ_R_ (cm^−1^) and µ (cm^−1^), the parameters of MFP (cm), HVL (cm), and TVL (cm) are derived according to Eqs. 3–5 as listed in Table 2.

**Table 6:** Parameters of fast neutron attenuation with statistical uncertainty.

Concrete type	Σ_R_ (cm^−1^)	MFP (cm)	HVL (cm)	TVL (cm)
CC	0.084 ± 0.003	11.90 ± 0.48	8.25 ± 0.33	27.41 ± 1.10
GC10	0.085 ± 0.003	11.76 ± 0.47	8.15 ± 0.33	27.09 ± 1.09
GC15	0.087 ± 0.003	11.49 ± 0.46	7.97 ± 0.32	26.47 ± 1.06

**Table 7:** Parameters of gamma-ray attenuation with statistical uncertainty.

Concrete type	µ (cm^−1^)	MFP (cm)	HVL (cm)	TVL (cm)
CC	0.060 ± 0.002	16.67 ± 0.67	11.55 ± 0.46	38.38 ± 1.54
GC10	0.049 ± 0.002	20.41 ± 0.82	14.15 ± 0.57	47.00 ± 1.88
GC15	0.045 ± 0.002	22.22 ± 0.89	15.40 ± 0.62	51.17 ± 2.05

#### Fast neutron attenuation

Concerning the fast neutron attenuation, Fig. 18 and Table 6 display the impact of GS on fast neutron shielding of concrete specimens with 10% and 15% GS by weight of PC (GC10 and GC15, respectively), with respect to the control mix (CC with 100% PC). It was found that the addition of GS marginally improves the fast neutron attenuation, with GC10 showing a 1.20% increase and GC15, showing a 3.50% increase compared to CC (Fig. 18 and Table 6). This can be verified by the improvement in the parameters of the fast neutron attenuation, including Σ_R_, MFP, HVL, and TVL as scheduled in Table 6. This can be ascribed to presence of light elements, primarily hydrogen and carbon, as indicated by the high LOI% (Table 3). These elements are found in minerals like graphite, talc, and actinolite. More specifically, these light elements can attenuate the fast neutrons through the elastic collision and absorption^99^. Otherwise, this enhancing effect of these elements on the attenuation surpasses the detrimental effect of GS on concrete density and porosity (Table 5), which explains the limited enhancing effect of GS on the fast neutron attenuation.

#### Gamma-ray attenuation

In contrast to its positive effect on fast neutron attenuation, the incorporation of GS at both 10% and 15% replacement levels exhibits a detrimental influence on gamma-ray attenuation (Fig. 19 and Table 7). Specifically, GC10 and GC15 reveal reductions in attenuation capacity by 18.80% and 25.90%, respectively, compared to CC. This could be due to the malignant impact of GS on the microstructure (Fig. 17c). More specifically, the higher proportion of unreactive cement particles combined with the reduced formation of hydration products leads to a less refined microstructure, which in turn deteriorates the gamma-ray attenuation capacity. In agreement with^110^, this porous microstructure may act as a potential pathway for radiation leakage, leading to reduced radiation attenuation. Also, the increased porosity observed in GC10 and GC15 further contributes to the deterioration in gamma-ray attenuation, as evidenced in Table 5. These findings can be confirmed by the parameters of gamma-rays, including µ, MFP, HVL, and TVL, as listed in Table 7.

## Limitations and future research

- To reduce the detrimental effect of GS, as a filler material, on concrete strength, it is recommended to reduce the w/b from 0.50 (as in the recent work) to lower values such as 0.35. Also, the utilization of superplasticizer (e.g., polycarboxylate) is suggested to diminish the excess mixing water. This lower w/b can optimize the packing of cementitious matrix and reduce the microcracking and then improve the mechanical and radiation shielding properties.
- In case of conducting lower w/b, it is necessary to assess the GS influence on concrete durability, regarding chloride ion penetration, sulfate resistance, and acid attack. These assessments will unveil new secrets of the GS material as a filler in the concrete.
- For improving the radiation shielding of GS, it can be combined as a filler material with heavyweight aggregates (barite, magnetite, and hematite) to balance neutron and γ-ray shielding.
- More processes can be applied to GS material for triggering its pozzolanic behaviour such as mechanical grinding to nanosized powder, alkali activation, calcination at 700 °C, or CO_2_ mineralization. These processes can transform the crystalline structure of GS to amorphous phases, activating its surfaces.
- The antifungal performance of concrete mixtures incorporating GS material can be evaluated in future studies.

## Conclusion

- Graphite schist (GS) is mainly composed of talc and actinolite with minor graphite, quartz, and muscovite. It exhibits fine particle size, high surface area, and stable thermal behavior up to 800 °C, highlighting its suitability in fire-resistant concrete.
- GS displayed superior inhibitory effects against several fungal strains compared with PC, suggesting an added value in applications where microbial resistance is desirable, especially in humid or biologically aggressive environments, such as wastewater treatment plants and sewage tunnels.
- GS functions primarily as a filler material with a lower pozzolanic reactivity rather than a reactive pozzolanic material. At 10% replacement (GC10), concrete retained long-term and sustained compressive strength values comparable to the CC, while 15% substitution (GC15) caused greater reductions. Extended curing mitigated strength loss, indicating prolonged hydration activity. Therefore, GS can be incorporated in non-structural or secondary concrete elements (pavements, blocks, precast panels), where ultra-high strength is not essential.
- 10% GS enhanced the particle packing of the cementitious matrix and ITZ. However, it resulted in the formation of unhydrated cement residues. Otherwise, 15% GS deteriorated the microstructure by forming microcracks in ITZ.
- GS slightly improved the fast-neutron attenuation of concrete, while it reduced the gamma-ray attenuation efficiency. This enhancement in fast-neutron attenuation is attributed to the presence of hydrogen- and carbon-bearing minerals in GS, such as talc, actinolite, and graphite. However, the relatively low density of GS increased the concrete porosity, which restricted the improvement in fast-neutron attenuation and negatively affected the gamma-ray shielding.

## Data Availability

Data will be available on request.
